# Supplementary material for: Study protocol: an investigation of the prevalence of autism among adults admitted to acute mental health wards: a cross-sectional pilot study
Source: BMJ Open. 2019 Dec 23;9(12):e033169. doi: 10.1136/bmjopen-2019-033169 (PMC7008424; doi:10.1136/bmjopen-2019-033169)
Supplement: Author's manuscript [file bmjopen-2019-033169.draft_revisions.pdf]

BMJ Open

BMJ Open is committed to open peer review. As part of this commitment we make the peer review history of every article we publish publicly available.

When an article is published we post the peer reviewers' comments and the authors' responses online. We also post the versions of the paper that were used during peer review. These are the versions that the peer review comments apply to.

The versions of the paper that follow are the versions that were submitted during the peer review process. They are not the versions of record or the final published versions. They should not be cited or distributed as the published version of this manuscript.

BMJ Open is an open access journal and the full, final, typeset and author-corrected version of record of the manuscript is available on our site with no access controls, subscription charges or pay-per-view fees (http://bmjopen.bmj.com).

If you have any questions on BMJ Open's open peer review process please email info.bmjopen@bmj.com

BMJ Open

Study Protocol: An investigation of the prevalence of autism among adults admitted to acute mental health wards: A cross-sectional study

Journal:	*BMJ Open*
Manuscript ID	bmjopen-2019-033169
Article Type:	Protocol
Date Submitted by the Author:	30-Jul-2019
Complete List of Authors:	Tromans, Sam; University of Leicester, Department of Health Sciences; Leicestershire Partnership NHS Trust, Learning Disability Psychiatry<br>Yao, Guiqing; University of Leicester Department of Health Sciences<br>Kiani, Reza; University of Leicester Department of Health Sciences; Leicestershire Partnership NHS Trust, Learning Disability Psychiatry<br>Alexander, Regi; University of Leicester Department of Health Sciences; Hertfordshire Partnership University NHS Foundation Trust<br>Al-Uzri, Mohammed; University of Leicester Department of Health Sciences; Leicestershire Partnership NHS Trust, General Adult Psychiatry<br>Brugha, Terry; University of Leicester Department of Health Sciences; Leicestershire Partnership NHS Trust, General Adult Psychiatry
Keywords:	Adult psychiatry < PSYCHIATRY, PUBLIC HEALTH, EPIDEMIOLOGY, MENTAL HEALTH, Autism

SCHOLARONE™
Manuscripts

Study Protocol: An investigation of the prevalence of autism among adults admitted to acute mental health wards: A cross-sectional study

Sam Tromans [1,2], Guiqing Yao [1], Reza Kiani [1,2], Regi Alexander [1,3], Mohammed Al-Uzri [1,2], Traolach Brugha [1,2]

[1] Department of Health Sciences, University of Leicester, George Davies Centre, University Road, Leicester, United Kingdom, LE1 7RH

[2] Leicestershire Partnership NHS Trust, Riverside House, Bridge Park Plaza, Bridge Park Road, Thurmaston, Leicester, United Kingdom, LE4 8PQ

[3] Hertfordshire Partnership University NHS Foundation Trust, The Colonnades, Beaconsfield Road, Hatfield, Hertfordshire, United Kingdom, AL10 8YE

Corresponding author:

Sam Tromans

Address: Room 3.06, Department of Health Sciences, University of Leicester, George Davies Centre, University Road, Leicester, United Kingdom, LE1 7RH

E-mail: sjt56@leicester.ac.uk

Telephone: 0116 373 6801

Fax: 0116 295 7250

Keywords:

Adult psychiatry, public health, epidemiology, mental health, autism.

Word count:

2,333 (excluding title page, abstract, references, figures and tables).

ABSTRACT

Introduction

Autism Spectrum Disorders (ASDs) are associated with difficulties in social interaction, communication and restricted, repetitive behaviours. Much is known about their community prevalence among adults, data on adult inpatients within an acute mental health setting is lacking.

This study aims to estimate the prevalence of ASDs among adults admitted to acute mental health wards, and examine the association between ASDs and psychiatric and physical comorbidities within this group.

Methods and Analysis

A multiple-phase approach will be used. Phase 1 will involve testing of 200 patients and corresponding informants, using the Autism Quotient (AQ), the informant version of the Social Responsiveness Scale, 2nd edition – Adult, the self and informant versions of the Adult Social Behaviour Questionnaire and the EuroQol-5D-5L. Patients with Intellectual Disability (ID) will bypass Phase 1.

Phase 2 will involve diagnostic testing of a subgroup of 40 patients with the Diagnostic Interview for Social and Communication Disorders, the Autism Diagnostic Observation Schedule version 2 and the ASD interview within the Schedules for Clinical Assessment in Neuropsychiatry version 3. 25±5 patients will not have ID and be selected via stratified random sampling according to AQ score; 15±5 patients will have ID. Phase 2 patients will be interviewed with the Physical Health Conditions and Mental Illness Diagnoses and Treatment sections of the 2014 Adult Psychiatric Morbidity Survey.

Prevalence estimates will be based on the proportion of Phase 2 participants who satisfy ICD-10-DCR and DSM-5 diagnostic criteria for ASD, adjusting for selection and non-response.

Univariate analysis will be conducted for comorbidities, to identify the level of their association with an ASD diagnosis.

Ethics and Dissemination:

Study oversight is provided by the University of Leicester. The NHS Health Research Authority have provided written approval. Study results will be disseminated via conference presentations and peer-reviewed publications.

Registration Details:

ISRCTN27739943

For peer review only

Strengths and Limitations of this Study

- The study is currently limited to a single site, limiting its generalisability. However, applications to extend to other sites are currently in progress.
- The planned sample size is in keeping with a feasibility study, though there are plans to expand to a larger trial upon completion.
- The primary outcome measure (meeting diagnostic criteria for ASDs) is somewhat contingent upon the diagnostic criteria used, though internationally recognised criteria have been used (the ICD-10-DCR and DSM-5).
- The study will be conducted under double-blind conditions, whereby neither patients nor health professionals conducting Phase 2 clinical assessments will have knowledge of the participants corresponding Phase 1 questionnaire results.
- The study design allows consideration of patients with and without intellectual disabilities separately, in contrast to many other studies of its type.

INTRODUCTION

ASDs are lifelong neurodevelopmental conditions characterised by impairments in reciprocal social interaction and communication, as well as restricted, stereotyped and repetitive behaviours.[1] These difficulties should manifest prior to three years of age, thus a detailed developmental history forms a key part of the diagnostic assessment process. Though originating in childhood, ASDs follow a persistent course throughout adult life.[2] Whilst the level of impairment associated with ASDs varies between individuals, the impact on both those with ASD and their families is always profound.[3]

The diagnostic criteria for ASD according to the ICD-10-DCR and DSM-5 diagnostic classification systems are summarized in Table 1. It should be noted that the transition from DSM-IV to DSM-5 criteria for ASD appears to reduce the number of individuals satisfying

diagnostic criteria, with only about 50-75% of individuals maintaining diagnoses, a phenomenon which disproportionately affects higher functioning non-intellectually disabled (non-ID) individuals.[4] However, the vast majority of what is known about ASD comes from research involving child populations, with under 15% of UK-based ASD research being focussed exclusively on adults,[5] with a similar focus on children seen internationally.[6]

When autism was first described in the medical literature, it was considered a rare condition.[9] However, there has been a significant rise in reported prevalence estimates in recent decades, which has been attributed to a myriad of factors, including broadening of diagnostic criteria, changes in study methodology, increasing knowledge of ASD among lay persons and professionals, and possibly a genuine increase in prevalence.[10]

ASDs represent a major global public health issue, responsible for over 111 Disability Adjusted Life Years per 100,000 persons.[11] Community-based prevalence rates for ASDs in adults within the general population show considerable variation across studies over time, though a recent large-scale epidemiological study estimated a prevalence rate of 1.1% (95% CI 0.3-1.9).[11,12]

A large United Kingdom-based study of 7,274 participants in the Adult Psychiatric Morbidity Survey (APMS)[13] coupled with a register of 290 adults with intellectual disability (ID),[1] found an overall estimated ASD prevalence of 1.1% (95% CI 0.3-1.9%).[12] This is in keeping with the findings of a child study of 56,946 participants by Baird *et al.,*[14] whom found an overall estimated prevalence of ASDs of 116.1 per 10,000 (95% CI 90.4-141.8). However, whilst the Brugha *et al.* [12] ASD prevalence findings for people with mild ID (1.0%, 95% CI 0.4-2.2) were similar to the overall findings, those with moderate to profound ID had

[1] Defined by DSM-5 as deficits in intellectual functioning (typified by an IQ score of ≤70) coupled with deficits or impairments of adaptive functioning, with onset during the developmental period.

For peer review only

a markedly increased risk of ASD, with prevalence rates of 42.3% (95% CI 31.1-54.3) and 35.2% (95% CI 23.5-49.0) in males and females respectively.

However, relative to the community setting, there has been very little research into the prevalence of ASDs among adults within acute mental health inpatient settings.[15] Previous work appears to suggest that the prevalence of ASDs could be significantly greater in this group than that of the general population and that they may be substantially underdiagnosed in this group, with prevalence estimates varying from 1.5 – 9.9%.[16-19]

The vast majority of research on psychiatric comorbidity in patients with ASDs has been conducted in child and adolescent populations.[20] Such studies have shown high rates of comorbid psychiatric conditions among individuals with ASDs, with rates ranging from 70.8% [21] to 80.9%,[22] and commonly comorbid conditions including anxiety disorders, obsessive compulsive disorders, phobias, oppositional defiant disorder and attention deficit hyperactivity disorder.[21-25] Notably, a study by Brereton *et al.* [25] found that children with ASDs experienced significantly higher levels of psychopathology compared to those with ID, a group which in itself is associated with an elevated risk of psychiatric disorder.[26]

However, it is unclear how the burden of psychiatric comorbidity seen in children with ASDs transitions into adult life.[27] It is essential to understand this natural course of ASDs and its comorbidities in order to develop appropriate services to meet the needs of this patient group.[28] Additionally, some psychiatric disorders, such as schizophrenia, do not typically manifest until late adolescence onwards,[29] so child comorbidity studies into such disorders would be of limited value.

A scoping review by Cashin *et al.* [30] found a lack of research pertaining to the physical health of individuals with diagnosed ASDs, and that most studies were limited to retrospective analysis of healthcare records rather than involving any form of direct health assessment. Additionally, these studies were focussed on individuals with previously

For peer review only

recognised ASD diagnosed through routine clinical assessment, rather than having been identified through population-based epidemiological studies. Current evidence suggests that children with ASDs are at greater risk of a range of comorbid physical illness compared to their non-ASD peers, including obesity,[31-33] epilepsy,[34,35] asthma,[35] sleep disorders,[36] and gastrointestinal disorders.[36] This assertion is further supported in a recent systematic review by Muskens *et al.,*[37] whom described medical disorders across a range of medical areas (including immunology, neurology and gastroenterology) as being widespread among children with ASD. However, despite the relative paucity of research in child populations, even less is known about the burden of physical comorbidity among adults with ASDs.[38]

The aims of this prospective study are to estimate the prevalence of ASDs amongst adults based on acute mental health inpatient units, as well as examining the association between ASDs and other psychiatric and physical comorbidities within this group.

METHODS AND ANALYSIS

Study Design

The study (also known as the SPRINT[2] study) will employ a multiple-phase cross-sectional design for participants without ID, and a single-phase design for participants with ID (Figure 1). A multiple-phase approach to studying ASD prevalence has been used in several previous similar studies,[12,19,39] as the ASD clinical interview-based diagnostic assessments are both time and resource intensive.[40] Thus, a multi-phase approach permits coverage of a larger study population per unit of resource. However, such an approach is heavily reliant on the accuracy of the tool used in the first phase in identifying probable cases of ASD.

[2] The Prevalence of Social Communication PRoblems in Adult Psychiatric INpaTients.

In terms of the multiple-phase design for non-ID patients, transition to Phase 2 will be selected via stratified random sampling according to the patient's scores on the Autism Quotient (AQ)[41] questionnaire (Table 2).

The rationale for a single-phase design in patients with ID is twofold. Firstly, there is a lack of validated and relevant ASD questionnaires for this patient group.[42] Secondly, the prevalence of ASD in this patient group is substantially higher in community studies,[12] such that a single-phase design would be less resource-inefficient in identifying individuals meeting ASD diagnostic criteria within this group.

Study Population

The total sample will comprise 200 patients between the ages of 18-65 years, with a history of having been a psychiatric inpatient on an adult acute mental health ward during the study time period (6th March 2019 to 31st December 2020). The inclusion and exclusion criteria are summarized in Table 3. Out of these 200 patients undergoing Phase 1 testing, 40 will transition to Phase 2 of the study.

Study participants will be recruited from two inpatient units based in Leicestershire, UK. These units serve a population of approximately 1 million people living in both urban and rural areas within Leicester, Leicestershire and Rutland.[43] Applications to extend the study to further sites are currently in progress.

Patient and Public Involvement

The study design was refined following consultation group sessions with both patients with ID and their carers, as well as patients without ID and their carers. Significant changes made to the study design were made as a result of these sessions, including reduction of the

questionnaire and interview burden, as well as incorporation of a stigma measure for patients with ID, as this was considered a research priority by patients with ID and their carers.

Patients and carers will all receive a report summarising the findings of the study, unless they request not to, and an easy-read version of the report will be developed for patients with ID.

Measures

The measures to be used are summarized in Table 4. The Phase 1 measures, which will be used to test non-ID participants only, include the AQ [41], which is recommended by the National Institute for Health and Care Excellence as referral tools for adults with possible ASD whom do not have ID [42]. Also included in the test battery are ASD tools which are less comprehensively validated in adult populations, but appear to show promise, including the Informant version of the Social Responsiveness Scale, 2nd edition [44] and the Self-report and Informant versions of the Adult Social Behaviour Questionnaire, ASBQ [45]. Additionally, both participants and informants will complete the EuroQol-5D-5L,[46] a self-report instrument that measures general health.

The Phase 2 measures include the DISCO [47] (though the interview will be limited to the diagnostic algorithm, psychiatric conditions and forensic problems items) and ADOS-2,[48] both of which are tools with established validity for the diagnosis of ASDs. Also, psychiatric and physical comorbidities will be assessed using the relevant sections of the 2014 APMS interview.[13] Other tests include the field testing of the ASD interview of version 3 of the Schedules for Clinical Assessment in Neuropsychiatry, as well as the Stigma Questionnaire for people with Intellectual Disability.[49]

For peer review only

Outcome Parameters

The primary outcome parameter is the presence or absence of meeting ICD-10-DCR and DSM-5 diagnostic criteria for ASDs respectively. Secondary outcome parameters include the presence or absence of comorbid psychiatric and physical health diagnoses.

Data Analysis Plan

The primary analysis will be based on patients who transition to Phase 2 of the study. The ASD prevalence estimate will be calculated based on the proportion of Phase 2 participants who meet the ICD-10-DCR and DSM-5 diagnostic criteria for ASD on clinical assessment, with adjustment for selection and non-response, as well as the different study designs for the ID and non-ID patient subgroups.

With regards to the comorbidity data, univariate analysis will be carried out for individual comorbidities, to identify the level of their association with meeting the diagnostic criteria for ASD, relative to those who do not meet such criteria, as well as corresponding OR, 95% CI and p-values.

Microsoft Excel, as well as a dedicated statistical software package – either SPSS or R – will be used to assist in data analysis.

Sample Size Justification

The sample size was calculated with direct statistician support, as well as the use of a statistical textbook and related software.[50] For a significance level of 0.05 and power of 85%, assuming estimates of 1% community ASD prevalence and 5% inpatient ASD prevalence,[15] a required sample size of 374 patients is required. Ideally, we would be subjecting a minimum of 374 patients to Phase 2 testing. This is because only participants completing Phase 2

interview assessments will yield data pertaining to the primary outcome variable (i.e. the presence of absence of satisfying diagnostic criteria for ASD).

However, in the first instance, around 40 patients (from the minimum of 200 subjected to Phase 1 testing) will be selected for Phase 2 testing (via stratified random sampling for non-ID patients, whereas ID patients will progress automatically to Phase 2). Our results will assume that the data for these 40 patients is representative of the larger group subjected to Phase 1 testing. Owing to the sample size, the SPRINT study should be considered a pilot/feasibility study, with a view towards sample size expansion upon its completion.

Ethics and Dissemination

The written approval of the National Health Service Health Research Authority has been obtained, as well as the local ethics committee of the study centre. Written informed consent is and will be obtained from every participant. Significant modifications to the study protocol will be communicated to relevant members of the research team. Any results from this study will be published in peer-reviewed journals and conference proceedings. Additionally, the study's findings will be disseminated to all participants, including easy-read versions of the findings for those with intellectual disabilities.

Contributor Statement

ST, TB, GY, RK, RA and MA-U were involved in drafting the study protocol. TB and ST conceived of the idea for this study. ST is the principal investigator for the study, under the supervision of TB. ST led in writing the manuscript, TB and GY contributed revisions, and all authors approved the final manuscript.

Competing Interests

TB is directly involved with the development of the ASD interview questions for the Schedules for Assessment in Neuropsychiatry version 3, which have not yet been formally published; this study is involved in testing the clinical utility of these questions. Additionally, TB and RK were authors of the 2014 APMS, from which two questionnaires are being used. Neither TB nor RK will be receiving any monetary payment as a result of the inclusion of the ASD interview questions or the 2014 APMS questionnaires within this study.

Funding

This study was supported by funding awarded by an NIHR Senior Investigator, as well as receiving funding and resource support from the NIHR, as a result of the study being adopted onto the NIHR Clinical Research Network Portfolio of studies.

Data Sharing Statement

Deidentified participant data will be made available upon reasonable request from Sam Tromans, contactable on sjt56@leicester.ac.uk. The time period for availability would be within 6 months of journal publication of the study findings. Protocols and statistical analysis plans would be similarly available.

REFERENCES

1. World Health Organization. *The ICD-10 classification of mental and behavioural disorders: Clinical descriptions and diagnostic guidelines.* World Health Organization: Geneva, 1992.

2. Howlin P. Outcome in adult life for more able individuals with autism or asperger syndrome. *Autism*. 2000;4:63-83.

3. Newschaffer CJ, Croen LA, Daniels J, et al. The epidemiology of autism spectrum disorders. *Annu Rev Public Health*. 2007;28:235-258.

4. Smith IC, Reichow B, Volkmar FR. The effects of DSM-5 criteria on number of individuals diagnosed with autism spectrum disorder: A systematic review. *J Autism Dev Disord*. 2015;45:2541-2552.

5. MacLeod A, Guldberg K, Kossyvaki L, *et al*. Mind the gap: What is missing in the autism research agenda? https://www.birmingham.ac.uk/research/perspective/autism-research-experts.aspx. (Accessed 12 July 2019).

6. Lever AG, Geurts HM. Psychiatric co-occurring symptoms and disorders in young, middle-aged, and older adults with autism spectrum disorder. *J Autism Dev Disord*. 2016;46:1916-1930.

7. World Health Organization. *The ICD-10 classification of mental and behavioural disorders: Diagnostic criteria for research.* World Health Organization: Geneva, 1993.

8. American Psychiatric Association. *Diagnostic and statistical manual of mental disorders: DSM-5.* American Psychiatric Publishing: Arlington, 2013.

9. Kanner L. Early infantile autism. *J Pediatr*. 1944; 25: 211-217.

10. Wing L, Potter D. The epidemiology of autistic spectrum disorders: Is the prevalence rising? *Dev Disabil Res Rev*. 2002;8:151-161.

For peer review only

11. Baxter A, Brugha T, Erskine H, *et al*. The epidemiology and global burden of autism spectrum disorders. *Psychol Med*. 2015;45:601-613.

12. Brugha TS, Spiers N, Bankart J, *et al.* Epidemiology of autism in adults across age groups and ability levels. *Br J Psychiatry*. 2016;209:498-503.

13. McManus S, Bebbington P, Jenkins R, *et al*. *Mental health and wellbeing in England: Adult psychiatric morbidity survey 2014.* NHS Digital: Leeds, 2016.

14. Baird G, Simonoff E, Pickles A, *et al.* Prevalence of disorders of the autism spectrum in a population cohort of children in south thames: The special needs and autism project (SNAP). *Lancet*. 2006;368:210-215.

15. Tromans S, Chester V, Kiani R, *et al*. The prevalence of autism spectrum disorders in adult psychiatric inpatients: A systematic review . *Clin Pract Epidemiol Ment Health*. 2018; 14: 177-187.

16. Mandell DS, Lawer LJ, Branch K, *et al.* Prevalence and correlates of autism in a state psychiatric hospital. *Autism*. 2012;16:557-567.

17. Shah A, Holmes N, Wing L. Prevalence of autism and related conditions in adults in a mental handicap hospital. *Appl Res Ment Retard*. 1982;3:303-317.

18. Scragg P, Shah A. Prevalence of asperger's syndrome in a secure hospital. *Br J Psychiatry*. 1994;165:679-682.

19. Hare DJ, Gould J, Mills R, *et al*. *A preliminary study of individuals with autistic spectrum disorders in three special hospitals in England.* National Autistic Society: London, 1999.

For peer review only

20. Ghaziuddin M, Zafar S. Psychiatric comorbidity of adults with autism spectrum disorders. *Clin Neuropsychiatry*. 2008;5:9-12.

21. Simonoff E, Pickles A, Charman T, *et al*. Psychiatric disorders in children with autism spectrum disorders: Prevalence, comorbidity, and associated factors in a population-derived sample. *J Am Acad Child Adolesc Psychiatry*. 2008;47:921-929.

22. de Bruin EI, Ferdinand RF, Meester S, *et al.* High rates of psychiatric co-morbidity in PDD-NOS. *J Autism Dev Disord*. 2007;37:877-886.

23. Mattila M, Hurtig T, Haapsamo H, *et al.* Comorbid psychiatric disorders associated with asperger syndrome/high-functioning autism: A community-and clinic-based study. *J Autism Dev Disord*. 2010;40:1080-1093.

24. Leyfer OT, Folstein SE, Bacalman S, *et al*. Comorbid psychiatric disorders in children with autism: Interview development and rates of disorders. *J Autism Dev Disord*. 2006;36:849-861.

25. Brereton AV, Tonge BJ, Einfeld SL. Psychopathology in children and adolescents with autism compared to young people with intellectual disability. *J Autism Dev Disord*. 2006;36:863-870.

26. Cooper SA, Smiley E, Morrison J, *et al*. Mental ill-health in adults with intellectual disabilities: Prevalence and associated factors. *Br J Psychiatry*. 2007;190:27-35.

27. Joshi G, Wozniak J, Petty C, *et al.* Psychiatric comorbidity and functioning in a clinically referred population of adults with autism spectrum disorders: A comparative study. *J Autism Dev Disord*. 2013;43:1314-1325.

For peer review only

28. Howlin P, Goode S, Hutton J *et al*. Adult outcome for children with autism. *J Child Psychol Psychiatry*. 2004;45:212-229.

29. Cascio MT, Cella M, Preti A, *et al*. Gender and duration of untreated psychosis: A systematic review and meta-analysis. *Early Interv Psychiatry*. 2012;6:115-127.

30. Cashin A, Buckley T, Trollor JN, *et al*. A scoping review of what is known of the physical health of adults with autism spectrum disorder. *J Intellect Disabil*. 2016;22:96-108.

31. Eaves LC, Ho HH. Young adult outcome of autism spectrum disorders. *J Autism Dev Disord*. 2008;38:739-747.

32. Ho HH, Eaves LC, Peabody D. Nutrient intake and obesity in children with autism. *Focus Autism Other Dev Disabl*. 1997;12:187-192.

33. Granich J, Lin A, Hunt A, *et al*. Obesity and associated factors in youth with an autism spectrum disorder. *Autism*. 2016;20:916-926.

34. Levy SE, Giarelli E, Lee LC, *et al.* Autism spectrum disorder and co-occurring developmental, psychiatric, and medical conditions among children in multiple populations of the united states. *J Dev Behav Pediatr*. 2010;31:267-275.

35. Schieve LA, Gonzalez V, Boulet SL, *et al.* Concurrent medical conditions and health care use and needs among children with learning and behavioral developmental disabilities, national health interview survey, 2006–2010. *Res Dev Disabil*. 2012;33:467-476.

36. Bauman ML. Medical comorbidities in autism: Challenges to diagnosis and treatment. *Neurotherapeutics*. 2010;7:320-327.

For peer review only

37. Muskens JB, Velders FP, Staal WG. Medical comorbidities in children and adolescents with autism spectrum disorders and attention deficit hyperactivity disorders: A systematic review. *Eur Child Adolesc Psychiatry*. 2017;26:1093-1103.

38. Croen LA, Zerbo O, Qian Y, *et al.* The health status of adults on the autism spectrum. *Autism*. 2015;19:814-823.

39. Tyrer F, Leaver A, Lewis S, *et al. Testing adults for possible social and communication disorders, including autism spectrum disorders.* NIHR CLAHRC Report: Leicester, 2013.

40. Ferriter M, Hare D, Bendall P, *et al.* Brief report: Assessment of a screening tool for autistic spectrum disorders in adult population. *J Autism Dev Disord*. 2001;31:351-353.

41. Baron-Cohen S, Wheelwright S, Skinner R, *et al*. The autism-spectrum quotient (AQ): Evidence from asperger syndrome/high-functioning autism, malesand females, scientists and mathematicians. *J Autism Dev Disord*. 2001;31:5-17.

42. National Institute for Health and Care Excellence. Autism spectrum disorder in adults: Diagnosis and management (CG142). https://www.nice.org.uk/guidance/cg142/resources/autism-spectrum-disorder-in-adults-diagnosis-and-management-pdf-35109567475909 (Accessed 12 July 2019).

43. Leicestershire Partnership NHS Trust. Basic facts and figures. . http://www.leicspart.nhs.uk/_Aboutus-WhoWeAreandWhatWeDo-FactsandFigures.aspx (Accessed 12 July 2019).

44. National Institute for Health and Care Excellence. Autism spectrum disorder in adults: Diagnosis and management. NICE guideline [CG142]. 2012.

For peer review only

44. Constantino JN, Gruber CP. *Social responsiveness scale (SRS).* Western Psychological Services: California, 2012.

45. Horwitz E, Schoevers R, Ketelaars C, *et al.* Clinical assessment of ASD in adults using self-and other-report: Psychometric properties and validity of the adult social behavior questionnaire (ASBQ). *Res Autism Spectr Disord*. 2016;24:17-28.

46. Herdman M, Gudex C, Lloyd A, *et al.* Development and preliminary testing of the new five-level version of EQ-5D (EQ-5D-5L). *Qual Life Res*. 2011;20:1727-1736.

47. Wing L, Leekam SR, Libby SJ, *et al*. The diagnostic interview for social and communication disorders: Background, inter-rater reliability and clinical use. *J Child Psychol Psychiatry*. 2002;43:307-325.

48. Lord C, Rutter M, DiLavore P *et al*. *Autism diagnostic observation schedule–2nd edition (ADOS-2).* Western Psychological Corporation: California, 2012.

49. Ali A, Strydom A, Hassiotis A, *et al*. A measure of perceived stigma in people with intellectual disability. *Br J Psychiatry*. 2008;193:410-415.

50. Machin D, Campbell MJ, Tan S, *et al*. *Sample size tables for clinical studies.* Wiley-Blackwell: New Jersey, 2011.

For peer review only

Table 1: Summaries of the ICD-10-DCR[7] and DSM-5[8] diagnostic criteria for ASD. For the diagnostic criteria in full, please refer to the source texts.

**ICD-10-DCR Criteria for Childhood Autism[3]:**<br>A. Presence of abnormal or impaired development before the age of three years<br>B. Qualitative abnormalities in reciprocal social interaction<br>C. Qualitative abnormalities in communication<br>D. Restricted, repetitive, and stereotyped patterns of behaviour, interests and activities<br>E. The clinical picture is not attributable to the other varieties of pervasive developmental disorder; specific developmental disorder of receptive language with secondary socio-emotional problems; reactive attachment disorder or disinhibited attachment disorder; mental retardation with some associated emotional or behavioural disorder; schizophrenia of unusually early onset; and Rett's syndrome.
**DSM-5 Criteria for Autism Spectrum Disorder:**<br>A. Persistent deficits in social communication and social interaction across multiple contexts<br>B. Restricted, repetitive patterns of behaviour, interests, or activities<br>C. Symptoms must be present in the early developmental period<br>D. Symptoms cause clinically significant impairment in social, occupational or other important areas of current functioning<br>E. These disturbances are not better explained by intellectual disability (ID)[4]

[3] ICD-10-DCR also recognises the diagnosis of 'atypical autism', whereby the features of abnormal development only manifest for the first time after 3 years of age (A) and/or the affected individual does not satisfy all three of the clinical diagnostic criteria (B-D).
[4] DSM-5 recognises that ASD and ID frequently co-exist, but that "to make comorbid diagnoses of autism spectrum disorder and intellectual disability, social communication should be below that expected for general developmental level 8."

For peer review only

Table 2: The probability of selection for Phase 2 according to total scores in the AQ questionnaire. This probability framework has been validated in a previous study conducted on adult participants from a mixture of inpatient and community mental health settings.[39] Participants scoring higher on these questionnaires will have a greater probability of selection.

AQ score	Probability of selection
≤19	0.1
20-24	0.2
25-29	0.3
30-39	0.6
≥40	1.0

For peer review only

Table 3: Inclusion and exclusion criteria. Additionally, participants lacking capacity will be withdrawn from the study if they become distressed by the assessment process and/or their guardians are not in agreement with them remaining in the study.

Inclusion Criteria	Exclusion Criteria
Aged between 18-65 years.	<18 or >65 years of age
Being or having been a psychiatric inpatient on an adult acute mental health ward during the study time period.	Having no history of being a psychiatric inpatient on an adult acute mental health ward during the study time period.
	Clinical diagnosis of dementia.
	Not understanding written and/or verbal English

For peer review only

Table 4: Summary of study measures

Measure	Purpose
**Phase 1** (Non-ID participants only)	
Autism Quotient, AQ [41]	Measuring likelihood of ASD.
Informant version of the Social Responsiveness Scale,2nd edition, SRS-A [44]	
Self-report and Informant versions of the Adult Social Behaviour Questionnaire, ASBQ [45]	
Self-report and Informant versions of the EuroQol-5D-5L, EQ-5D-5L (Herdman et al., 2011)	Measuring quality of life.
**Phase 2**	
Diagnostic Interview for Social and Communication Disorders, DISCO [47]	Establishing whether participant meets diagnostic criteria for ASDs.
Autism Diagnostic Observation Schedule version 2, ADOS-2 [48]	
The Mental Illness Diagnoses and Treatment section of the 2014 Adult Psychiatric Morbidity Survey, APMS [13]	Establishing participant's psychiatric and physical health comorbidities.
The Physical Health Conditions section of the 2014 APMS [13]	
ASD interview subsection of version 3 of the Schedules for Clinical Assessment in Neuropsychiatry, ASD-SCAN-3	Field-testing of interview schedule (for non-ID participants only)
Stigma Questionnaire for people with Intellectual Disability, SQID [49]	Participants experience of stigma (for ID participants only)

For peer review only

Figure 1: Flow chart summary of study design.

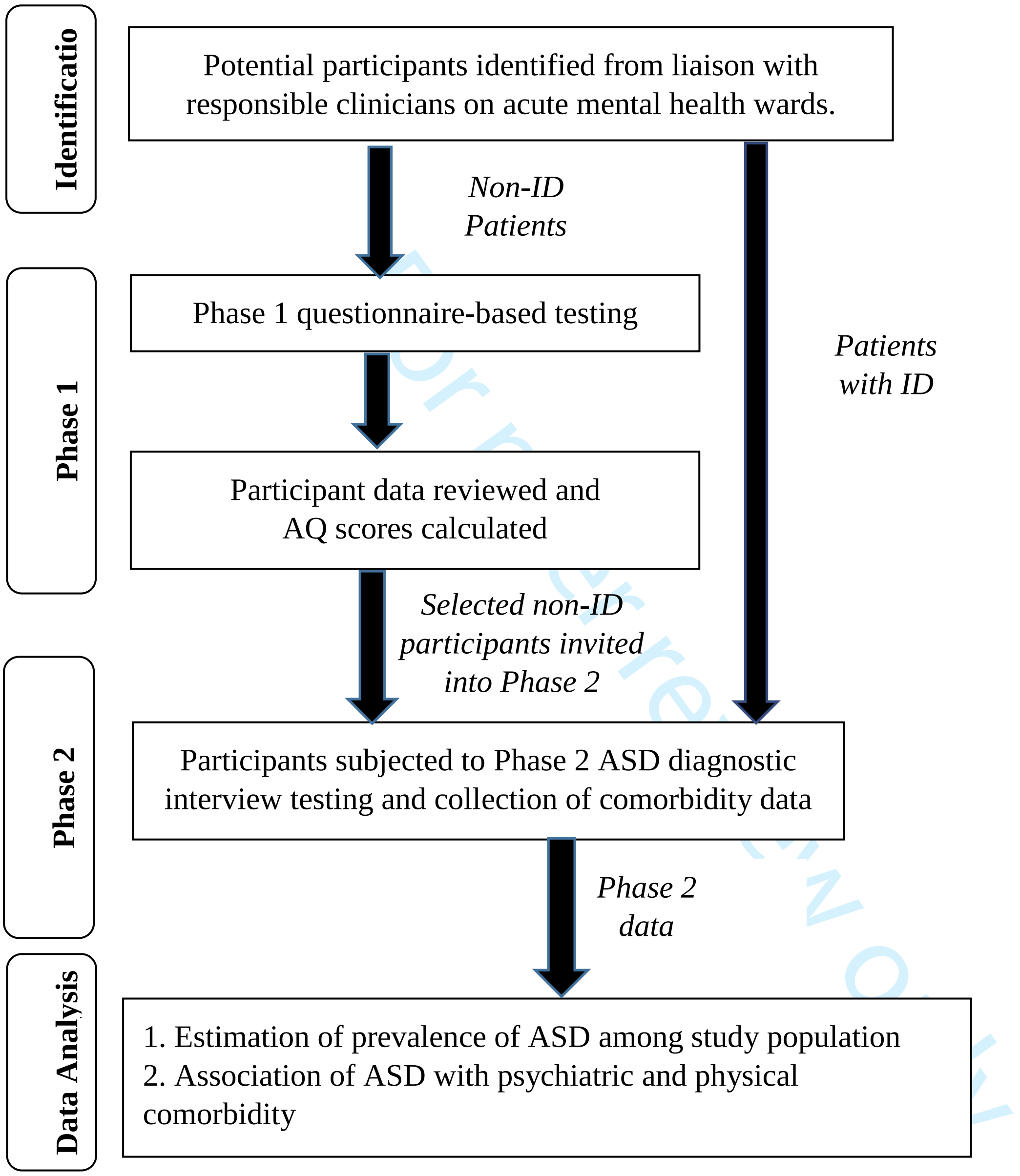

Acknowledgements

The authors would like to acknowledge the following individuals for their contributions to the study. Nicola Spiers, Maria Viskaduraki and Zoe Morgan for statistical support. Elizabeta Mukaetova-Ladinska and Verity Chester for assistance with the systematic reviews underpinning the study. Dave Clarke and Henry Simkins for assistance with study design. Natalie Marking, Hannah Harrison, Tom Pringle, Deborah Glancy, Joanna McGarr, Kris Roberts and Sam Pollen for support with study recruitment. Joanne Prosser and Naishali Chandarana for advice and support in developing easy-read materials. Freya Tyrer and Lesley Thoms for assistant with Patient and Public Involvement Sessions. The members of the Peoples Forum (Leicester, United Kingdom) and patients and carers at Leicestershire Partnership NHS Trust for providing valued input in the Patient and Public Involvement Sessions.

ew only

STROBE Statement—checklist of items that should be included in reports of observational studies

	Item No	Recommendation
**Title and abstract**	1	(*a*) Indicate the study's design with a commonly used term in the title or the abstract
		(*b*) Provide in the abstract an informative and balanced summary of what was done and what was found — X n/a (study protocol)
**Introduction**		
Background/rationale	2	Explain the scientific background and rationale for the investigation being reported
Objectives	3	State specific objectives, including any prespecified hypotheses
**Methods**		
Study design	4	Present key elements of study design early in the paper
Setting	5	Describe the setting, locations, and relevant dates, including periods of recruitment, exposure, follow-up, and data collection
Participants	6	(*a*) *Cohort study*—Give the eligibility criteria, and the sources and methods of selection of participants. Describe methods of follow-up<br>*Case-control study*—Give the eligibility criteria, and the sources and methods of case ascertainment and control selection. Give the rationale for the choice of cases and controls<br>*Cross-sectional study*—Give the eligibility criteria, and the sources and methods of selection of participants
		(*b*) *Cohort study*—For matched studies, give matching criteria and number of exposed and unexposed<br>*Case-control study*—For matched studies, give matching criteria and the number of controls per case
Variables	7	Clearly define all outcomes, exposures, predictors, potential confounders, and effect modifiers. Give diagnostic criteria, if applicable
Data sources/ measurement	8*	For each variable of interest, give sources of data and details of methods of assessment (measurement). Describe comparability of assessment methods if there is more than one group
Bias	9	Describe any efforts to address potential sources of bias
Study size	10	Explain how the study size was arrived at
Quantitative variables	11	Explain how quantitative variables were handled in the analyses. If applicable, describe which groupings were chosen and why
Statistical methods	12	(*a*) Describe all statistical methods, including those used to control for confounding
		(*b*) Describe any methods used to examine subgroups and interactions
		(*c*) Explain how missing data were addressed
		(*d*) *Cohort study*—If applicable, explain how loss to follow-up was addressed<br>*Case-control study*—If applicable, explain how matching of cases and controls was addressed<br>*Cross-sectional study*—If applicable, describe analytical methods taking account of sampling strategy
		(*e*) Describe any sensitivity analyses

Continued on next page

**Results**		
Participants	13*	(a) Report numbers of individuals at each stage of study—eg numbers potentially eligible, examined for eligibility, confirmed eligible, included in the study, completing follow-up, and analysed
		(b) Give reasons for non-participation at each stage
		(c) Consider use of a flow diagram
Descriptive data	14*	(a) Give characteristics of study participants (eg demographic, clinical, social) and information on exposures and potential confounders
		(b) Indicate number of participants with missing data for each variable of interest
		(c) *Cohort study*—Summarise follow-up time (eg, average and total amount)
Outcome data	15*	*Cohort study*—Report numbers of outcome events or summary measures over time
		Case-control study—Report numbers in each exposure category, or summary measures of exposure
		Cross-sectional study—Report numbers of outcome events or summary measures
Main results	16	(*a*) Give unadjusted estimates and, if applicable, confounder-adjusted estimates and their precision (eg, 95% confidence interval). Make clear which confounders were adjusted for and why they were included
		(*b*) Report category boundaries when continuous variables were categorized
		(*c*) If relevant, consider translating estimates of relative risk into absolute risk for a meaningful time period
Other analyses	17	Report other analyses done—eg analyses of subgroups and interactions, and sensitivity analyses
**Discussion**		
Key results	18	Summarise key results with reference to study objectives
Limitations	19	Discuss limitations of the study, taking into account sources of potential bias or imprecision. Discuss both direction and magnitude of any potential bias
Interpretation	20	Give a cautious overall interpretation of results considering objectives, limitations, multiplicity of analyses, results from similar studies, and other relevant evidence
Generalisability	21	Discuss the generalisability (external validity) of the study results
**Other information**		
Funding	22	Give the source of funding and the role of the funders for the present study and, if applicable, for the original study on which the present article is based

*Give information separately for cases and controls in case-control studies and, if applicable, for exposed and unexposed groups in cohort and cross-sectional studies.

Note: An Explanation and Elaboration article discusses each checklist item and gives methodological background and published examples of transparent reporting. The STROBE checklist is best used in conjunction with this article (freely available on the Web sites of PLoS Medicine at http://www.plosmedicine.org/, Annals of Internal Medicine at http://www.annals.org/, and Epidemiology at http://www.epidem.com/). Information on the STROBE Initiative is available at www.strobe-statement.org.

BMJ Open

Study Protocol: An investigation of the prevalence of autism among adults admitted to acute mental health wards: A cross-sectional study

Journal:	*BMJ Open*
Manuscript ID	bmjopen-2019-033169.R1
Article Type:	Protocol
Date Submitted by the Author:	17-Nov-2019
Complete List of Authors:	Tromans, Sam; University of Leicester, Department of Health Sciences; Leicestershire Partnership NHS Trust, Learning Disability Psychiatry<br>Yao, Guiqing; University of Leicester Department of Health Sciences<br>Kiani, Reza; University of Leicester Department of Health Sciences; Leicestershire Partnership NHS Trust, Learning Disability Psychiatry<br>Alexander, Regi; University of Leicester Department of Health Sciences; Hertfordshire Partnership University NHS Foundation Trust<br>Al-Uzri, Mohammed; University of Leicester Department of Health Sciences; Leicestershire Partnership NHS Trust, General Adult Psychiatry<br>Brugha, Traolach; University of Leicester Department of Health Sciences; Leicestershire Partnership NHS Trust, General Adult Psychiatry
**Primary Subject Heading**:	Mental health
Secondary Subject Heading:	Public health, Epidemiology
Keywords:	Adult psychiatry < PSYCHIATRY, PUBLIC HEALTH, EPIDEMIOLOGY, MENTAL HEALTH, Autism

SCHOLARONE™
Manuscripts

Study Protocol: An investigation of the prevalence of autism among adults admitted to acute mental health wards: A cross-sectional study

Sam Tromans [1,2], Guiqing Yao [1], Reza Kiani [1,2], Regi Alexander [1,3], Mohammed Al-Uzri [1,2], Traolach Brugha [1,2]

[1] Department of Health Sciences, University of Leicester, George Davies Centre, University Road, Leicester, United Kingdom, LE1 7RH

[2] Leicestershire Partnership NHS Trust, Riverside House, Bridge Park Plaza, Bridge Park Road, Thurmaston, Leicester, United Kingdom, LE4 8PQ

[3] Hertfordshire Partnership University NHS Foundation Trust, The Colonnades, Beaconsfield Road, Hatfield, Hertfordshire, United Kingdom, AL10 8YE

Corresponding author:

Sam Tromans

Address: Room 3.06, Department of Health Sciences, University of Leicester, George Davies Centre, University Road, Leicester, United Kingdom, LE1 7RH

E-mail: sjt56@leicester.ac.uk

Telephone: 0116 373 6801

Fax: 0116 295 7250

Keywords:

Adult psychiatry, public health, epidemiology, mental health, autism.

Word count:

2,508 (excluding title page, abstract, references, figures and tables).

For peer review only

ABSTRACT

Introduction

Autism Spectrum Disorders (ASDs) are associated with difficulties in social interaction, communication and restricted, repetitive behaviours. Much is known about their community prevalence among adults, data on adult inpatients within an acute mental health setting is lacking.

This study aims to estimate the prevalence of ASDs among adults admitted to acute mental health wards, and examine the association between ASDs and psychiatric and physical comorbidities within this group.

Methods and Analysis

A multiple-phase approach will be used. Phase 1 will involve testing of 200 patients and corresponding informants, using the Autism Quotient (AQ), the informant version of the Social Responsiveness Scale, 2nd edition – Adult, the self and informant versions of the Adult Social Behaviour Questionnaire and the EuroQol-5D-5L. Patients with Intellectual Disability (ID) will bypass Phase 1.

Phase 2 will involve diagnostic testing of a subgroup of 40 patients with the Diagnostic Interview for Social and Communication Disorders, the Autism Diagnostic Observation Schedule version 2 and the ASD interview within the Schedules for Clinical Assessment in Neuropsychiatry version 3. 25±5 patients will not have ID and be selected via stratified random sampling according to AQ score; 15±5 patients will have ID. Phase 2 patients will be interviewed with the Physical Health Conditions and Mental Illness Diagnoses and Treatment sections of the 2014 Adult Psychiatric Morbidity Survey.

Prevalence estimates will be based on the proportion of Phase 2 participants who satisfy ICD-10-DCR and DSM-5 diagnostic criteria for ASD, adjusting for selection and non-response.

Univariate analysis will be conducted for comorbidities, to identify the level of their association with an ASD diagnosis.

Ethics and Dissemination:

Study oversight is provided by the University of Leicester. The NHS Health Research Authority have provided written approval. Study results will be disseminated via conference presentations and peer-reviewed publications.

Registration Details:

ISRCTN27739943

For peer review only

Strengths and Limitations of this Study

- The study is currently limited to a single site, limiting its generalisability. However, applications to extend to other sites are currently in progress.
- The primary outcome measure (meeting diagnostic criteria for ASDs) is somewhat contingent upon the diagnostic criteria used, though internationally recognised criteria have been used (the ICD-10-DCR and DSM-5).
- The study will be conducted under double-blind conditions, whereby neither patients nor health professionals conducting Phase 2 clinical assessments will have knowledge of the participants corresponding Phase 1 questionnaire results.
- The study design allows consideration of patients with and without intellectual disabilities separately, in contrast to many other studies of its type.

INTRODUCTION

ASDs are lifelong neurodevelopmental conditions characterised by impairments in reciprocal social interaction and communication, as well as restricted, stereotyped and repetitive behaviours.[1] These difficulties should manifest prior to three years of age, thus a detailed developmental history forms a key part of the diagnostic assessment process. Though originating in childhood, ASDs follow a persistent course throughout adult life.[2] Whilst the level of impairment associated with ASDs varies between individuals, the impact on both those with ASD and their families is always profound.[3]

The diagnostic criteria for ASD according to the ICD-10-DCR[4] and DSM-5[5] diagnostic classification systems are summarized in Table 1. It should be noted that the transition from DSM-IV to DSM-5 criteria for ASD appears to reduce the number of individuals satisfying diagnostic criteria, with only about 50-75% of individuals maintaining diagnoses, a phenomenon which disproportionately affects higher functioning non-

intellectually disabled (non-ID) individuals.[6] However, the vast majority of what is known about ASD comes from research involving child populations, with under 15% of UK-based ASD research being focussed exclusively on adults,[7] with a similar focus on children seen internationally.[8]

When autism was first described in the medical literature, it was considered a rare condition.[9] However, there has been a significant rise in reported prevalence estimates in recent decades, which has been attributed to a myriad of factors, including broadening of diagnostic criteria, changes in study methodology, increasing knowledge of ASD among lay persons and professionals, and possibly a genuine increase in prevalence.[10]

ASDs represent a major global public health issue, responsible for over 111 Disability Adjusted Life Years per 100,000 persons.[11] Community-based prevalence rates for ASDs in adults within the general population show considerable variation across studies over time, though a recent large-scale epidemiological study estimated a prevalence rate of 1.1% (95% CI 0.3-1.9).[11,12]

A large United Kingdom-based study of 7,274 participants in the 2014 Adult Psychiatric Morbidity Survey (APMS)[13] coupled with a register of 290 adults with intellectual disability (ID),[1] found an overall estimated ASD prevalence of 1.1% (95% CI 0.3-1.9%).[12] This is in keeping with the findings of a child study of 56,946 participants by Baird *et al.,*[14] who found an overall estimated prevalence of ASDs of 116.1 per 10,000 (95% CI 90.4-141.8). However, whilst the Brugha *et al.*[12] ASD prevalence findings for people with mild ID (1.0%, 95% CI 0.4-2.2) were similar to the overall findings, those with moderate to

[1] Defined by DSM-5 as deficits in intellectual functioning (typified by an IQ score of ≤70) coupled with deficits or impairments of adaptive functioning, with onset during the developmental period.

For peer review only

profound ID had a markedly increased risk of ASD, with prevalence rates of 42.3% (95% CI 31.1-54.3) and 35.2% (95% CI 23.5-49.0) in males and females respectively.

However, relative to the community setting, there has been very little research into the prevalence of ASDs among adults within acute mental health inpatient settings.[15] Previous work appears to suggest that the prevalence of ASDs could be significantly greater in this group than that of the general population and that they may be substantially underdiagnosed in this group, with prevalence estimates varying from 1.5 – 9.9%.[16-19]

The vast majority of research on psychiatric comorbidity in patients with ASDs has been conducted in child and adolescent populations.[20] Such studies have shown high rates of comorbid psychiatric conditions among individuals with ASDs, with rates ranging from 70.8% [21] to 80.9%,[22] and commonly comorbid conditions including anxiety disorders, obsessive compulsive disorders, phobias, oppositional defiant disorder and attention deficit hyperactivity disorder.[21-25] Notably, a study by Brereton *et al.*[25] found that children with ASDs experienced significantly higher levels of psychopathology compared to those with ID, a group which in itself is associated with an elevated risk of psychiatric disorder.[26]

However, it is unclear how the burden of psychiatric comorbidity seen in children with ASDs transitions into adult life.[27] It is essential to understand this natural course of ASDs and its comorbidities in order to develop appropriate services to meet the needs of this patient group.[28] Additionally, some psychiatric disorders, such as schizophrenia, do not typically manifest until late adolescence onwards,[29] so child comorbidity studies into such disorders would be of limited value.

A scoping review by Cashin *et al.*[30] found a lack of research pertaining to the physical health of individuals with diagnosed ASDs, and that most studies were limited to retrospective analysis of healthcare records rather than involving any form of direct health assessment. Additionally, these studies were focussed on individuals with previously

For peer review only

recognised ASD diagnosed through routine clinical assessment, rather than having been identified through population-based epidemiological studies. Current evidence suggests that children with ASDs are at greater risk of a range of comorbid physical illness compared to their non-ASD peers, including obesity,[31-33] epilepsy,[34,35] asthma,[35] sleep disorders,[36] and gastrointestinal disorders.[36] This assertion is further supported in a recent systematic review by Muskens *et al.,*[37] who described medical disorders across a range of medical areas (including immunology, neurology and gastroenterology) as being widespread among children with ASD. However, despite the relative paucity of research in child populations, even less is known about the burden of physical comorbidity among adults with ASDs.[38]

The aims of this prospective study are to estimate the prevalence of ASDs amongst adults based on acute mental health inpatient units, as well as examining the association between ASDs and other psychiatric and physical comorbidities within this group.

METHODS AND ANALYSIS

Study Design

The study (also known as the SPRINT[2] study) will employ a multiple-phase cross-sectional design for participants without ID, and a single-phase design for participants with ID (Figure 1). A multiple-phase approach to studying ASD prevalence has been used in several previous similar studies,[12,19,39] as the ASD clinical interview-based diagnostic assessments are both time and resource intensive.[40] Thus, a multi-phase approach permits coverage of a larger study population per unit of resource. However, such an approach is heavily reliant on the accuracy of the tool used in the first phase in identifying probable cases of ASD.

[2] The Prevalence of <u>S</u>ocial Communication <u>PR</u>oblems in Adult Psychiatric <u>IN</u>pa<u>T</u>ients.

In terms of the multiple-phase design for non-ID patients, transition to Phase 2 will be selected via stratified random sampling according to the patient's scores on the Autism Quotient (AQ)[41] questionnaire (Table 2). This weighted approach enables generation of a prevalence estimate for the population as a whole (compared to if there were a single threshold AQ score for Phase 2 progression), and has been extensively validated, including in the 2014 APMS.[13]

The rationale for a single-phase design in patients with ID is twofold. Firstly, there is a lack of validated and relevant ASD questionnaires for this patient group.[42] Secondly, the prevalence of ASD in this patient group is substantially higher in community studies,[12] such that a single-phase design would be less resource-inefficient in identifying individuals meeting ASD diagnostic criteria within this group.

Study Population

The total sample will comprise 200 patients between the ages of 18-65 years, with a history of having been a psychiatric inpatient on an adult acute mental health ward during the study time period (6th March 2019 to 31st December 2020). The inclusion and exclusion criteria are summarized in Table 3. Out of these 200 patients undergoing Phase 1 testing, 40 will transition to Phase 2 of the study. The Phase 2 patient group will consist of 25±5 non-ID patients, and 15±5 patients with ID.

Study participants will be recruited from two inpatient units based in Leicestershire, UK. These units serve a population of approximately 1 million people living in both urban and rural areas within Leicester, Leicestershire and Rutland.[43] Applications to extend the study to further sites are currently in progress.

Patient and Public Involvement

The study design was refined following consultation group sessions with both patients with ID and their carers, as well as patients without ID and their carers. Significant changes made to the study design were made as a result of these sessions, including reduction of the questionnaire and interview burden, as well as incorporation of a stigma measure for patients with ID, as this was considered a research priority by patients with ID and their carers.

Patients and carers will all receive a report summarising the findings of the study, unless they request not to, and an easy-read version of the report will be developed for patients with ID.

Measures

The measures to be used are summarized in Table 4. The Phase 1 measures, which will be used to test non-ID participants only, include the AQ, [41] which is recommended by the National Institute for Health and Care Excellence as referral tools for adults with possible ASD who do not have ID.[44] Also included in the test battery are ASD tools which are less comprehensively validated in adult populations, but appear to show promise, including the Informant version of the Social Responsiveness Scale, 2nd edition[45] and the Self-report and Informant versions of the Adult Social Behaviour Questionnaire, ASBQ.[46] Phase 1 also includes a basic information form for participants, including demographic details, encompassing basic demographic information, and information pertaining to mental and physical health, including the 2014 APMS mental illness and physical health conditions checklists, thus enabling measurement harmonisation and wider comparison.[13] Additionally, both participants and informants will complete the EuroQol-5D-5L,[47] a self-report instrument that measures general health.

The Phase 2 measures include the DISCO[48] (though the interview will be limited to the diagnostic algorithm, psychiatric conditions and forensic problems items) and ADOS-2,[49]

both of which are tools with established validity for the diagnosis of ASDs. Also, psychiatric and physical comorbidities will be assessed using the relevant sections of the 2014 APMS interview.[13] Other tests include the field testing of the ASD interview of version 3 of the Schedules for Clinical Assessment in Neuropsychiatry, as well as the Stigma Questionnaire for people with Intellectual Disability.[50]

Outcome Parameters

The primary outcome parameter is the presence or absence of meeting ICD-10-DCR and DSM-5 diagnostic criteria for ASDs respectively. Secondary outcome parameters, including the study phase in which the corresponding parameters are collected, are summarised in Table 5.

Data Analysis Plan

The primary outcome parameter will be based on patients who transition to Phase 2 of the study. The ASD prevalence estimate will be calculated based on the proportion of Phase 2 participants who meet the ICD-10-DCR and DSM-5 diagnostic criteria for ASD on clinical assessment, with adjustment for selection and non-response, as well as the different study designs for the ID and non-ID patient subgroups.

For the feasibility study, the analyses will be descriptive. For continuous variables, values and standard deviations will be reported; for categorical variables, percentages and standard deviations will be reported. We will use multiple imputation to account for any missing data. For the full-powered study, the generalised linear model will be used to estimate the difference between groups, as well as corresponding OR, 95% CI and p-values.

For peer review only - http://bmjopen.bmj.com/site/about/guidelines.xhtml

The analyses will be primarily conducted in SPSS, but for more advanced statistical analyses (such as use of the generalised linear model), and graphical representation, we will use R.

Sample Size Justification

The sample size was calculated with direct statistician support, as well as the use of a statistical textbook and related software.[51] For a significance level of 0.05 and power of 85%, assuming estimates of 1% community ASD prevalence and 5% inpatient ASD prevalence,[15] a sample size of 374 patients is required. Ideally, we would be subjecting a minimum of 374 patients to Phase 2 testing. This is because only participants completing Phase 2 interview assessments will yield data pertaining to the primary outcome variable (i.e. the presence of absence of satisfying diagnostic criteria for ASD).

However, in the first instance, around 40 patients (from the minimum of 200 subjected to Phase 1 testing) will be selected for Phase 2 testing (via stratified random sampling for non-ID patients, whereas ID patients will progress automatically to Phase 2). The Phase 1 sample size of 200 patients takes into account the probability sampling method for Phase 2 selection, in addition to allowing for losses to follow-up for Phase 1 participants invited for Phase 2 testing. Our results will assume that the data for these 40 patients is representative of the larger group subjected to Phase 1 testing. Owing to the sample size, the SPRINT study should be considered a pilot/feasibility study, the intention of which being to establish and further refine the technical, administrative and logistical aspects of the study, with a view towards sample size expansion upon its completion. The sample size represents an appropriate sample size for a feasibility study of this type.[52,53]

Ethics and Dissemination

The written approval of the National Health Service Health Research Authority has been obtained, as well as the local ethics committee of the study centre. Written informed consent is and will be obtained from every participant. Significant modifications to the study protocol will be communicated to relevant members of the research team. Any results from this study will be published in peer-reviewed journals and conference proceedings. Additionally, the study's findings will be disseminated to all participants, including easy-read versions of the findings for those with intellectual disabilities.

Contributor Statement

ST, TB, GY, RK, RA and MA-U were involved in drafting the study protocol. TB and ST conceived of the idea for this study. ST is the principal investigator for the study, under the supervision of TB. ST led in writing the manuscript, TB and GY contributed revisions, and all authors approved the final manuscript.

Competing Interests

TB is directly involved with the development of the ASD interview questions for the Schedules for Assessment in Neuropsychiatry version 3, which have not yet been formally published; this study is involved in testing the clinical utility of these questions. Additionally, TB and RK were authors of the 2014 APMS, from which two questionnaires are being used. Neither TB nor RK will be receiving any monetary payment as a result of the inclusion of the ASD interview questions or the 2014 APMS questionnaires within this study.

Funding

This study was supported by funding awarded by an NIHR Senior Investigator, as well as receiving funding and resource support from the NIHR, as a result of the study being adopted onto the NIHR Clinical Research Network Portfolio of studies.

Data Sharing Statement

Deidentified participant data will be made available upon reasonable request from Sam Tromans, contactable on sjt56@leicester.ac.uk. The time period for availability would be within 6 months of journal publication of the study findings. Protocols and statistical analysis plans would be similarly available.

REFERENCES

1. World Health Organization. *The ICD-10 classification of mental and behavioural disorders: Clinical descriptions and diagnostic guidelines.* World Health Organization: Geneva, 1992.

2. Howlin P. Outcome in adult life for more able individuals with autism or asperger syndrome. *Autism*. 2000;4:63-83.

3. Newschaffer CJ, Croen LA, Daniels J, et al. The epidemiology of autism spectrum disorders. *Annu Rev Public Health*. 2007;28:235-258.

4. World Health Organization. *The ICD-10 classification of mental and behavioural disorders: Diagnostic criteria for research.* World Health Organization: Geneva, 1993.

5. American Psychiatric Association. *Diagnostic and statistical manual of mental disorders: DSM-5.* American Psychiatric Publishing: Arlington, 2013.

For peer review only

6. Smith IC, Reichow B, Volkmar FR. The effects of DSM-5 criteria on number of individuals diagnosed with autism spectrum disorder: A systematic review. *J Autism Dev Disord*. 2015;45:2541-2552.

7. MacLeod A, Guldberg K, Kossyvaki L, *et al*. Mind the gap: What is missing in the autism research agenda? https://www.birmingham.ac.uk/research/perspective/autism-research-experts.aspx. (Accessed 12 July 2019).

8. Lever AG, Geurts HM. Psychiatric co-occurring symptoms and disorders in young, middle-aged, and older adults with autism spectrum disorder. *J Autism Dev Disord*. 2016;46:1916-1930.

9. Kanner L. Early infantile autism. *J Pediatr*. 1944; 25: 211-217.

10. Wing L, Potter D. The epidemiology of autistic spectrum disorders: Is the prevalence rising? *Dev Disabil Res Rev*. 2002;8:151-161.

11. Baxter A, Brugha T, Erskine H, *et al*. The epidemiology and global burden of autism spectrum disorders. *Psychol Med*. 2015;45:601-613.

12. Brugha TS, Spiers N, Bankart J, *et al.* Epidemiology of autism in adults across age groups and ability levels. *Br J Psychiatry*. 2016;209:498-503.

13. McManus S, Bebbington P, Jenkins R, *et al*. *Mental health and wellbeing in England: Adult psychiatric morbidity survey 2014.* NHS Digital: Leeds, 2016.

14. Baird G, Simonoff E, Pickles A, *et al.* Prevalence of disorders of the autism spectrum in a population cohort of children in south thames: The special needs and autism project (SNAP). *Lancet*. 2006;368:210-215.

For peer review only

15. Tromans S, Chester V, Kiani R, *et al*. The prevalence of autism spectrum disorders in adult psychiatric inpatients: A systematic review . *Clin Pract Epidemiol Ment Health*. 2018; 14: 177-187.

16. Mandell DS, Lawer LJ, Branch K, *et al.* Prevalence and correlates of autism in a state psychiatric hospital. *Autism*. 2012;16:557-567.

17. Shah A, Holmes N, Wing L. Prevalence of autism and related conditions in adults in a mental handicap hospital. *Appl Res Ment Retard*. 1982;3:303-317.

18. Scragg P, Shah A. Prevalence of asperger's syndrome in a secure hospital. *Br J Psychiatry*. 1994;165:679-682.

19. Hare DJ, Gould J, Mills R, *et al*. *A preliminary study of individuals with autistic spectrum disorders in three special hospitals in England.* National Autistic Society: London, 1999.

20. Ghaziuddin M, Zafar S. Psychiatric comorbidity of adults with autism spectrum disorders. *Clin Neuropsychiatry*. 2008;5:9-12.

21. Simonoff E, Pickles A, Charman T, *et al*. Psychiatric disorders in children with autism spectrum disorders: Prevalence, comorbidity, and associated factors in a population-derived sample. *J Am Acad Child Adolesc Psychiatry*. 2008;47:921-929.

22. de Bruin EI, Ferdinand RF, Meester S, *et al.* High rates of psychiatric co-morbidity in PDD-NOS. *J Autism Dev Disord*. 2007;37:877-886.

23. Mattila M, Hurtig T, Haapsamo H, *et al.* Comorbid psychiatric disorders associated with asperger syndrome/high-functioning autism: A community-and clinic-based study. *J Autism Dev Disord*. 2010;40:1080-1093.

For peer review only

24. Leyfer OT, Folstein SE, Bacalman S, *et al.* Comorbid psychiatric disorders in children with autism: Interview development and rates of disorders. *J Autism Dev Disord*. 2006;36:849-861.

25. Brereton AV, Tonge BJ, Einfeld SL. Psychopathology in children and adolescents with autism compared to young people with intellectual disability. *J Autism Dev Disord*. 2006;36:863-870.

26. Cooper SA, Smiley E, Morrison J, *et al*. Mental ill-health in adults with intellectual disabilities: Prevalence and associated factors. *Br J Psychiatry*. 2007;190:27-35.

27. Joshi G, Wozniak J, Petty C, *et al*. Psychiatric comorbidity and functioning in a clinically referred population of adults with autism spectrum disorders: A comparative study. *J Autism Dev Disord*. 2013;43:1314-1325.

28. Howlin P, Goode S, Hutton J *et al*. Adult outcome for children with autism. *J Child Psychol Psychiatry*. 2004;45:212-229.

29. Cascio MT, Cella M, Preti A, *et al*. Gender and duration of untreated psychosis: A systematic review and meta-analysis. *Early Interv Psychiatry*. 2012;6:115-127.

30. Cashin A, Buckley T, Trollor JN, *et al*. A scoping review of what is known of the physical health of adults with autism spectrum disorder. *J Intellect Disabil*. 2016;22:96-108.

31. Eaves LC, Ho HH. Young adult outcome of autism spectrum disorders. *J Autism Dev Disord*. 2008;38:739-747.

32. Ho HH, Eaves LC, Peabody D. Nutrient intake and obesity in children with autism. *Focus Autism Other Dev Disabl*. 1997;12:187-192.

For peer review only

33. Granich J, Lin A, Hunt A, *et al*. Obesity and associated factors in youth with an autism spectrum disorder. *Autism*. 2016;20:916-926.

34. Levy SE, Giarelli E, Lee LC, *et al.* Autism spectrum disorder and co-occurring developmental, psychiatric, and medical conditions among children in multiple populations of the united states. *J Dev Behav Pediatr*. 2010;31:267-275.

35. Schieve LA, Gonzalez V, Boulet SL, *et al.* Concurrent medical conditions and health care use and needs among children with learning and behavioral developmental disabilities, national health interview survey, 2006–2010. *Res Dev Disabil*. 2012;33:467-476.

36. Bauman ML. Medical comorbidities in autism: Challenges to diagnosis and treatment. *Neurotherapeutics*. 2010;7:320-327.

37. Muskens JB, Velders FP, Staal WG. Medical comorbidities in children and adolescents with autism spectrum disorders and attention deficit hyperactivity disorders: A systematic review. *Eur Child Adolesc Psychiatry*. 2017;26:1093-1103.

38. Croen LA, Zerbo O, Qian Y, *et al.* The health status of adults on the autism spectrum. *Autism*. 2015;19:814-823.

39. Brugha T, Tyrer F, Leaver A, *et al.* Testing adults by questionnaire for social and communication disorders, including autism spectrum disorders, in an adult mental health service population. *Int J Methods Psychiatr Res*. In press.

40. Ferriter M, Hare D, Bendall P, *et al.* Brief report: Assessment of a screening tool for autistic spectrum disorders in adult population. *J Autism Dev Disord*. 2001;31:351-353.

For peer review only

41. Baron-Cohen S, Wheelwright S, Skinner R, *et al*. The autism-spectrum quotient (AQ): Evidence from asperger syndrome/high-functioning autism, males and females, scientists and mathematicians. *J Autism Dev Disord*. 2001;31:5-17.

42. National Institute for Health and Care Excellence. Autism spectrum disorder in adults: Diagnosis and management (CG142). https://www.nice.org.uk/guidance/cg142/resources/autism-spectrum-disorder-in-adults-diagnosis-and-management-pdf-35109567475909 (Accessed 12 July 2019).

43. Leicestershire Partnership NHS Trust. Basic facts and figures. . http://www.leicspart.nhs.uk/_Aboutus-WhoWeAreandWhatWeDo-FactsandFigures.aspx (Accessed 12 July 2019).

44. National Institute for Health and Care Excellence. Autism spectrum disorder in adults: Diagnosis and management. NICE guideline [CG142]. 2012.

45. Constantino JN, Gruber CP. *Social responsiveness scale (SRS).* Western Psychological Services: California, 2012.

46. Horwitz E, Schoevers R, Ketelaars C, *et al.* Clinical assessment of ASD in adults using self-and other-report: Psychometric properties and validity of the adult social behavior questionnaire (ASBQ). *Res Autism Spectr Disord*. 2016;24:17-28.

47. Herdman M, Gudex C, Lloyd A, *et al.* Development and preliminary testing of the new five-level version of EQ-5D (EQ-5D-5L). *Qual Life Res*. 2011;20:1727-1736.

48. Wing L, Leekam SR, Libby SJ, *et al*. The diagnostic interview for social and communication disorders: Background, inter-rater reliability and clinical use. *J Child Psychol Psychiatry*. 2002;43:307-325.

For peer review only

49. Lord C, Rutter M, DiLavore P *et al*. *Autism diagnostic observation schedule–2nd edition (ADOS-2).* Western Psychological Corporation: California, 2012.

50. Ali A, Strydom A, Hassiotis A, *et al*. A measure of perceived stigma in people with intellectual disability. *Br J Psychiatry*. 2008;193:410-415.

51. Machin D, Campbell MJ, Tan S, *et al*. *Sample size tables for clinical studies.* Wiley-Blackwell: New Jersey, 2011.

52. Johanson GA, Brookes, GP. Initial scale development: sample size for pilot studies. *Educ Psychol Meas.* 2010;70:394-400.

53. Hertzog MA. Considerations in determining sample size for pilot studies. *Res Nurs Health.* 2008;31:180-191.

For peer review only

Table 1: Summaries of the ICD-10-DCR[4] and DSM-5[5] diagnostic criteria for ASD. For the diagnostic criteria in full, please refer to the source texts.

ICD-10-DCR Criteria for Childhood Autism[3]:

A. Presence of abnormal or impaired development before the age of three years
B. Qualitative abnormalities in reciprocal social interaction
C. Qualitative abnormalities in communication
D. Restricted, repetitive, and stereotyped patterns of behaviour, interests and activities
E. The clinical picture is not attributable to the other varieties of pervasive developmental disorder; specific developmental disorder of receptive language with secondary socio-emotional problems; reactive attachment disorder or disinhibited attachment disorder; mental retardation with some associated emotional or behavioural disorder; schizophrenia of unusually early onset; and Rett's syndrome.

DSM-5 Criteria for Autism Spectrum Disorder:

A. Persistent deficits in social communication and social interaction across multiple contexts
B. Restricted, repetitive patterns of behaviour, interests, or activities
C. Symptoms must be present in the early developmental period
D. Symptoms cause clinically significant impairment in social, occupational or other important areas of current functioning
E. These disturbances are not better explained by intellectual disability (ID)[4]

[3] ICD-10-DCR also recognises the diagnosis of ‘atypical autism’, whereby the features of abnormal development only manifest for the first time after 3 years of age (A) and/or the affected individual does not satisfy all three of the clinical diagnostic criteria (B-D).
[4] DSM-5 recognises that ASD and ID frequently co-exist, but that “to make comorbid diagnoses of autism spectrum disorder and intellectual disability, social communication should be below that expected for general developmental level.”

For peer review only - http://bmjopen.bmj.com/site/about/guidelines.xhtml

Table 2: The probability of selection for Phase 2 according to total scores in the AQ questionnaire. This probability framework has been validated in a previous study conducted on adult participants from a mixture of inpatient and community mental health settings.[39] Participants scoring higher on these questionnaires will have a greater probability of selection.

AQ score	Probability of selection
≤19	0.1
20-24	0.2
25-29	0.3
30-39	0.6
≥40	1.0

For peer review only

Table 3: Inclusion and exclusion criteria. Additionally, participants lacking capacity will be withdrawn from the study if they become distressed by the assessment process and/or their guardians are not in agreement with them remaining in the study.

**Inclusion Criteria**	**Exclusion Criteria**
Aged between 18-65 years.	<18 or >65 years of age
Being or having been a psychiatric inpatient on an adult acute mental health ward during the study time period.	Having no history of being a psychiatric inpatient on an adult acute mental health ward during the study time period.
	Clinical diagnosis of dementia.
	Not understanding written and/or verbal English

For peer review only

Table 4: Summary of study measures

Measure	Purpose
**Phase 1** (Non-ID participants only)	
Autism Quotient, AQ[41]	Measuring likelihood of ASD.
Informant version of the Social Responsiveness Scale,2nd edition, SRS-A[45]	
Self-report and Informant versions of the Adult Social Behaviour Questionnaire, ASBQ[46]	
Self-report and Informant versions of the EuroQol-5D-5L, EQ-5D-5L[47]	Measuring quality of life.
Basic information form	Collecting information pertaining to patient demographics, as well as the 2014 Adult Psychiatric Morbidity Survey (APMS) mental illness and physical health conditions checklists[13]
**Phase 2**	
Diagnostic Interview for Social and Communication Disorders, DISCO[48]	Establishing whether participant meets diagnostic criteria for ASDs.
Autism Diagnostic Observation Schedule version 2, ADOS-2[49]	
The Mental Illness Diagnoses and Treatment section of the 2014 APMS[13]	Establishing participant's psychiatric and physical health comorbidities.
The Physical Health Conditions section of the 2014 APMS[13]	
ASD interview subsection of version 3 of the Schedules for Clinical Assessment in Neuropsychiatry, ASD-SCAN-3	Field-testing of interview schedule (for non-ID participants only)
Stigma Questionnaire for people with Intellectual Disability, SQID[50]	Participants experience of stigma (for ID participants only)
Basic information form	Collecting information pertaining to patient demographics, as well as the 2014 Adult Psychiatric Morbidity Survey (APMS) mental illness and physical health conditions checklists[13] (for ID participants only, as this form is completed in Phase 1 for non-ID participants).

Table 5: Summary of secondary outcome parameters

Outcome Category	Corresponding Parameters	Study Phase in which Data is Collected
**Autism questionnaire data**	• Autism Quotient, AQ[41]<br>• Informant version of the Social Responsiveness Scale,2nd edition, SRS-A[45]<br>• Self-report and Informant versions of the Adult Social Behaviour Questionnaire, ASBQ[46]	• Phase 1
**Basic demographic information**	• Date of birth<br>• Sex<br>• Postcode<br>• Ethnic group<br>• Employment status<br>• Relationship status	• Phase 1 (non-ID participants)<br>• Phase 2 (ID participants)
**Mental health history**	• Date of most recent psychiatric hospital admission<br>• Discharge date of most recent psychiatric hospital admission (where applicable)<br>• Total number of inpatient psychiatric admissions<br>• 2014 Adult Psychiatric Morbidity Survey (APMS) mental illness checklist[13]	• Phase 1 (non-ID participants)<br>• Phase 2 (ID participants)
	• The Mental Illness Diagnoses and Treatment section of the 2014 APMS[13]	• Phase 2
**Physical health history**	• 2014 APMS physical health conditions checklist[13]	• Phase 1 (non-ID participants)<br>• Phase 2 (ID participants)
	• The Physical Health Conditions section of the 2014 APMS[13]	• Phase 2
**General health**	• EuroQol-5D-5L[47] data for participants and informants	• Phase 1
**Stigma experience (ID participants only)**	• Stigma Questionnaire for people with Intellectual Disability, SQID[50]	• Phase 2 (ID participants)

For peer review only

Figure 1: Flow chart summary of study design.

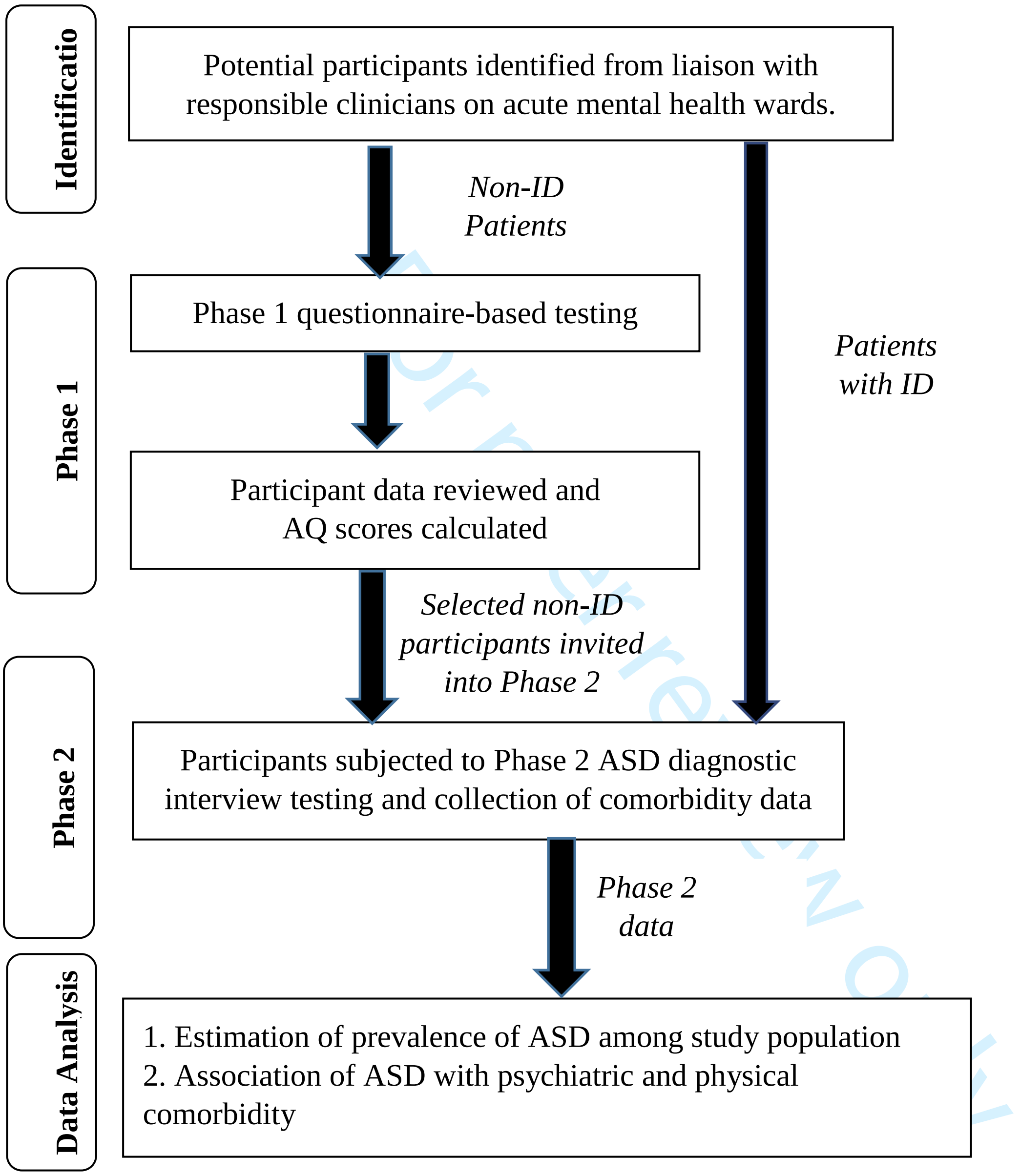

Acknowledgements

The authors would like to acknowledge the following individuals for their contributions to the study. Nicola Spiers, Maria Viskaduraki and Zoe Morgan for statistical support. Elizabeta Mukaetova-Ladinska and Verity Chester for assistance with the systematic reviews underpinning the study. Dave Clarke and Henry Simkins for assistance with study design. Natalie Marking, Hannah Harrison, Tom Pringle, Deborah Glancy, Joanna McGarr, Rebekah Pole, Kris Roberts and Sam Pollen for support with study recruitment. Joanne Prosser and Naishali Chandarana for advice and support in developing easy-read materials. Freya Tyrer and Lesley Thoms for assistant with Patient and Public Involvement Sessions. Debra Bugler, Janice Holmes, Joy Fellows and Robin Oxley-Boyle for administrative support. The members of the Peoples Forum (Leicester, United Kingdom) and patients and carers at Leicestershire Partnership NHS Trust for providing valued input in the Patient and Public Involvement Sessions.

For peer review only

STROBE Statement—checklist of items that should be included in reports of observational studies

	Item No	Recommendation
**Title and abstract**	1	(*a*) Indicate the study's design with a commonly used term in the title or the abstract
		(*b*) Provide in the abstract an informative and balanced summary of what was done and what was found — X n/a (study protocol
**Introduction**		
Background/rationale	2	Explain the scientific background and rationale for the investigation being reported
Objectives	3	State specific objectives, including any prespecified hypotheses
**Methods**		
Study design	4	Present key elements of study design early in the paper
Setting	5	Describe the setting, locations, and relevant dates, including periods of recruitment, exposure, follow-up, and data collection
Participants	6	(*a*) *Cohort study*—Give the eligibility criteria, and the sources and methods of selection of participants. Describe methods of follow-up<br>*Case-control study*—Give the eligibility criteria, and the sources and methods of case ascertainment and control selection. Give the rationale for the choice of cases and controls<br>*Cross-sectional study*—Give the eligibility criteria, and the sources and methods of selection of participants
		(*b*) *Cohort study*—For matched studies, give matching criteria and number of exposed and unexposed<br>*Case-control study*—For matched studies, give matching criteria and the number of controls per case
Variables	7	Clearly define all outcomes, exposures, predictors, potential confounders, and effect modifiers. Give diagnostic criteria, if applicable
Data sources/ measurement	8*	For each variable of interest, give sources of data and details of methods of assessment (measurement). Describe comparability of assessment methods if there is more than one group
Bias	9	Describe any efforts to address potential sources of bias
Study size	10	Explain how the study size was arrived at
Quantitative variables	11	Explain how quantitative variables were handled in the analyses. If applicable, describe which groupings were chosen and why
Statistical methods	12	(*a*) Describe all statistical methods, including those used to control for confounding
		(*b*) Describe any methods used to examine subgroups and interactions
		(*c*) Explain how missing data were addressed
		(*d*) *Cohort study*—If applicable, explain how loss to follow-up was addressed<br>*Case-control study*—If applicable, explain how matching of cases and controls was addressed<br>*Cross-sectional study*—If applicable, describe analytical methods taking account of sampling strategy
		(*e*) Describe any sensitivity analyses

Continued on next page

For peer review only - http://bmjopen.bmj.com/site/about/guidelines.xhtml

**Results**		
Participants	13*	(a) Report numbers of individuals at each stage of study—eg numbers potentially eligible, examined for eligibility, confirmed eligible, included in the study, completing follow-up, and analysed
		(b) Give reasons for non-participation at each stage
		(c) Consider use of a flow diagram
Descriptive data	14*	(a) Give characteristics of study participants (eg demographic, clinical, social) and information on exposures and potential confounders
		(b) Indicate number of participants with missing data for each variable of interest
		(c) *Cohort study*—Summarise follow-up time (eg, average and total amount)
Outcome data	15*	*Cohort study*—Report numbers of outcome events or summary measures over time
		Case-control study—Report numbers in each exposure category, or summary measures of exposure
		Cross-sectional study—Report numbers of outcome events or summary measures
Main results	16	(*a*) Give unadjusted estimates and, if applicable, confounder-adjusted estimates and their precision (eg, 95% confidence interval). Make clear which confounders were adjusted for and why they were included
		(*b*) Report category boundaries when continuous variables were categorized
		(*c*) If relevant, consider translating estimates of relative risk into absolute risk for a meaningful time period
Other analyses	17	Report other analyses done—eg analyses of subgroups and interactions, and sensitivity analyses
**Discussion**		
Key results	18	Summarise key results with reference to study objectives
Limitations	19	Discuss limitations of the study, taking into account sources of potential bias or imprecision. Discuss both direction and magnitude of any potential bias
Interpretation	20	Give a cautious overall interpretation of results considering objectives, limitations, multiplicity of analyses, results from similar studies, and other relevant evidence
Generalisability	21	Discuss the generalisability (external validity) of the study results
**Other information**		
Funding	22	Give the source of funding and the role of the funders for the present study and, if applicable, for the original study on which the present article is based

*Give information separately for cases and controls in case-control studies and, if applicable, for exposed and unexposed groups in cohort and cross-sectional studies.

Note: An Explanation and Elaboration article discusses each checklist item and gives methodological background and published examples of transparent reporting. The STROBE checklist is best used in conjunction with this article (freely available on the Web sites of PLoS Medicine at http://www.plosmedicine.org/, Annals of Internal Medicine at http://www.annals.org/, and Epidemiology at http://www.epidem.com/). Information on the STROBE Initiative is available at www.strobe-statement.org.

BMJ Open

Study Protocol: An investigation of the prevalence of autism among adults admitted to acute mental health wards: A cross-sectional pilot study

Journal:	*BMJ Open*
Manuscript ID	bmjopen-2019-033169.R2
Article Type:	Protocol
Date Submitted by the Author:	04-Dec-2019

Complete List of Authors:	Tromans, Sam; University of Leicester, Department of Health Sciences; Leicestershire Partnership NHS Trust, Learning Disability Psychiatry<br>Yao, Guiqing; University of Leicester Department of Health Sciences<br>Kiani, Reza; University of Leicester Department of Health Sciences; Leicestershire Partnership NHS Trust, Learning Disability Psychiatry<br>Alexander, Regi; University of Leicester Department of Health Sciences; Hertfordshire Partnership University NHS Foundation Trust<br>Al-Uzri, Mohammed; University of Leicester Department of Health Sciences; Leicestershire Partnership NHS Trust, General Adult Psychiatry<br>Brugha, Traolach; University of Leicester Department of Health Sciences; Leicestershire Partnership NHS Trust, General Adult Psychiatry

**Primary Subject Heading**:	Mental health
Secondary Subject Heading:	Public health, Epidemiology
Keywords:	Adult psychiatry < PSYCHIATRY, PUBLIC HEALTH, EPIDEMIOLOGY, MENTAL HEALTH, Autism

SCHOLARONE™
Manuscripts

Study Protocol: An investigation of the prevalence of autism among adults admitted to acute mental health wards: A cross-sectional pilot study

Sam Tromans [1,2], Guiqing Yao [1], Reza Kiani [1,2], Regi Alexander [1,3], Mohammed Al-Uzri [1,2], Traolach Brugha [1,2]

[1] Department of Health Sciences, University of Leicester, George Davies Centre, University Road, Leicester, United Kingdom, LE1 7RH

[2] Leicestershire Partnership NHS Trust, Riverside House, Bridge Park Plaza, Bridge Park Road, Thurmaston, Leicester, United Kingdom, LE4 8PQ

[3] Hertfordshire Partnership University NHS Foundation Trust, The Colonnades, Beaconsfield Road, Hatfield, Hertfordshire, United Kingdom, AL10 8YE

Corresponding author:

Sam Tromans

Address: Room 3.06, Department of Health Sciences, University of Leicester, George Davies Centre, University Road, Leicester, United Kingdom, LE1 7RH

E-mail: sjt56@leicester.ac.uk

Telephone: 0116 373 6801

Fax: 0116 295 7250

Keywords:

Adult psychiatry, public health, epidemiology, mental health, autism.

Word count:

2,675 (excluding title page, abstract, references, figures and tables).

For peer review only

ABSTRACT

Introduction

Autism Spectrum Disorders (ASDs) are associated with difficulties in social interaction, communication and restricted, repetitive behaviours. Much is known about their community prevalence among adults, data on adult inpatients within an acute mental health setting is lacking.

This study aims to estimate the prevalence of ASDs among adults admitted to acute mental health wards, and examine the association between ASDs and psychiatric and physical comorbidities within this group.

Methods and Analysis

A multiple-phase approach will be used. Phase 1 will involve testing of 200 patients and corresponding informants, using the Autism Quotient (AQ), the informant version of the Social Responsiveness Scale, 2nd edition – Adult, the self and informant versions of the Adult Social Behaviour Questionnaire and the EuroQol-5D-5L. Patients with Intellectual Disability (ID) will bypass Phase 1.

Phase 2 will involve diagnostic testing of a subgroup of 40 patients with the Diagnostic Interview for Social and Communication Disorders, the Autism Diagnostic Observation Schedule version 2 and the ASD interview within the Schedules for Clinical Assessment in Neuropsychiatry version 3. 25±5 patients will not have ID and be selected via stratified random sampling according to AQ score; 15±5 patients will have ID. Phase 2 patients will be interviewed with the Physical Health Conditions and Mental Illness Diagnoses and Treatment sections of the 2014 Adult Psychiatric Morbidity Survey.

Prevalence estimates will be based on the proportion of Phase 2 participants who satisfy ICD-10-DCR and DSM-5 diagnostic criteria for ASD, adjusting for selection and non-response.

Univariate analysis will be conducted for comorbidities, to identify the level of their association with an ASD diagnosis.

Ethics and Dissemination:

Study oversight is provided by the University of Leicester. The NHS Health Research Authority have provided written approval. Study results will be disseminated via conference presentations and peer-reviewed publications.

Registration Details:

ISRCTN27739943

For peer review only

Strengths and Limitations of this Study

- The study is currently limited to a single site, limiting its generalisability. However, applications to extend to other sites are currently in progress.
- The planned sample size is in keeping with a pilot study, though there are plans to expand to a larger trial upon completion.
- The primary outcome measure (meeting diagnostic criteria for ASDs) is somewhat contingent upon the diagnostic criteria used, though internationally recognised criteria have been used (the ICD-10-DCR and DSM-5).
- The study will be conducted under double-blind conditions, whereby neither patients nor health professionals conducting Phase 2 clinical assessments will have knowledge of the participants corresponding Phase 1 questionnaire results.
- The study design allows consideration of patients with and without intellectual disabilities separately, in contrast to many other studies of its type.

INTRODUCTION

ASDs are lifelong neurodevelopmental conditions characterised by impairments in reciprocal social interaction and communication, as well as restricted, stereotyped and repetitive behaviours.[1] These difficulties should manifest prior to three years of age, thus a detailed developmental history forms a key part of the diagnostic assessment process. Though originating in childhood, ASDs follow a persistent course throughout adult life.[2] Whilst the level of impairment associated with ASDs varies between individuals, the impact on both those with ASD and their families is always profound.[3]

The diagnostic criteria for ASD according to the ICD-10-DCR[4] and DSM-5[5] diagnostic classification systems are summarized in Table 1. It should be noted that the transition from DSM-IV to DSM-5 criteria for ASD appears to reduce the number of

individuals satisfying diagnostic criteria, with only about 50-75% of individuals maintaining diagnoses, a phenomenon which disproportionately affects higher functioning non-intellectually disabled (non-ID) individuals.[6] However, the vast majority of what is known about ASD comes from research involving child populations, with under 15% of UK-based ASD research being focussed exclusively on adults,[7] with a similar focus on children seen internationally.[8]

When autism was first described in the medical literature, it was considered a rare condition.[9] However, there has been a significant rise in reported prevalence estimates in recent decades, which has been attributed to a myriad of factors, including broadening of diagnostic criteria, changes in study methodology, increasing knowledge of ASD among lay persons and professionals, and possibly a genuine increase in prevalence.[10]

ASDs represent a major global public health issue, responsible for over 111 Disability Adjusted Life Years per 100,000 persons.[11] Community-based prevalence rates for ASDs in adults within the general population show considerable variation across studies over time, though a recent large-scale epidemiological study estimated a prevalence rate of 1.1% (95% CI 0.3-1.9).[11,12]

A large United Kingdom-based study of 7,274 participants in the 2014 Adult Psychiatric Morbidity Survey (APMS)[13] coupled with a register of 290 adults with intellectual disability (ID),[1] found an overall estimated ASD prevalence of 1.1% (95% CI 0.3-1.9%).[12] This is in keeping with the findings of a child study of 56,946 participants by Baird *et al.,*[14] who found an overall estimated prevalence of ASDs of 116.1 per 10,000 (95% CI 90.4-141.8). However, whilst the Brugha *et al.*[12] ASD prevalence findings for people with mild ID (1.0%, 95% CI 0.4-2.2) were similar to the overall findings, those with moderate to

[1] Defined by DSM-5 as deficits in intellectual functioning (typified by an IQ score of ≤70) coupled with deficits or impairments of adaptive functioning, with onset during the developmental period.

For peer review only

profound ID had a markedly increased risk of ASD, with prevalence rates of 42.3% (95% CI 31.1-54.3) and 35.2% (95% CI 23.5-49.0) in males and females respectively.

However, relative to the community setting, there has been very little research into the prevalence of ASDs among adults within acute mental health inpatient settings.[15] Previous work appears to suggest that the prevalence of ASDs could be significantly greater in this group than that of the general population and that they may be substantially underdiagnosed in this group, with prevalence estimates varying from 1.5 – 9.9%.[16-19]

The vast majority of research on psychiatric comorbidity in patients with ASDs has been conducted in child and adolescent populations.[20] Such studies have shown high rates of comorbid psychiatric conditions among individuals with ASDs, with rates ranging from 70.8% [21] to 80.9%,[22] and commonly comorbid conditions including anxiety disorders, obsessive compulsive disorders, phobias, oppositional defiant disorder and attention deficit hyperactivity disorder.[21-25] Notably, a study by Brereton *et al.*[25] found that children with ASDs experienced significantly higher levels of psychopathology compared to those with ID, a group which in itself is associated with an elevated risk of psychiatric disorder.[26]

However, it is unclear how the burden of psychiatric comorbidity seen in children with ASDs transitions into adult life.[27] It is essential to understand this natural course of ASDs and its comorbidities in order to develop appropriate services to meet the needs of this patient group.[28] Additionally, some psychiatric disorders, such as schizophrenia, do not typically manifest until late adolescence onwards,[29] so child comorbidity studies into such disorders would be of limited value.

A scoping review by Cashin *et al.*[30] found a lack of research pertaining to the physical health of individuals with diagnosed ASDs, and that most studies were limited to retrospective analysis of healthcare records rather than involving any form of direct health assessment. Additionally, these studies were focussed on individuals with previously

For peer review only

recognised ASD diagnosed through routine clinical assessment, rather than having been identified through population-based epidemiological studies. Current evidence suggests that children with ASDs are at greater risk of a range of comorbid physical illness compared to their non-ASD peers, including obesity,[31-33] epilepsy,[34,35] asthma,[35] sleep disorders,[36] and gastrointestinal disorders.[36] This assertion is further supported in a recent systematic review by Muskens *et al.,*[37] who described medical disorders across a range of medical areas (including immunology, neurology and gastroenterology) as being widespread among children with ASD. However, despite the relative paucity of research in child populations, even less is known about the burden of physical comorbidity among adults with ASDs.[38]

The aims of this prospective pilot study are to estimate the prevalence of ASDs amongst adults based on acute mental health inpatient units, as well as examining the association between ASDs and other psychiatric and physical comorbidities within this group.

METHODS AND ANALYSIS

Study Design

The study (also known as the SPRINT[2] study) will employ a multiple-phase cross-sectional design for participants without ID, and a single-phase design for participants with ID (Figure 1). A multiple-phase approach to studying ASD prevalence has been used in several previous similar studies,[12,19,39] as the ASD clinical interview-based diagnostic assessments are both time and resource intensive.[40] Thus, a multi-phase approach permits coverage of a larger study population per unit of resource. However, such an approach is heavily reliant on the accuracy of the tool used in the first phase in identifying probable cases of ASD.

[2] The Prevalence of Social Communication PRoblems in Adult Psychiatric INpaTients.

In terms of the multiple-phase design for non-ID patients, transition to Phase 2 will be selected via stratified random sampling according to the patient's scores on the Autism Quotient (AQ)[41] questionnaire (Table 2). This weighted approach enables generation of a prevalence estimate for the population as a whole (compared to if there were a single threshold AQ score for Phase 2 progression), and has been extensively validated, including in the 2014 APMS.[13]

The rationale for a single-phase design in patients with ID is twofold. Firstly, there is a lack of validated and relevant ASD questionnaires for this patient group.[42] Secondly, the prevalence of ASD in this patient group is substantially higher in community studies,[12] such that a single-phase design would be less resource-inefficient in identifying individuals meeting ASD diagnostic criteria within this group.

Study Population

The total sample will comprise 200 patients between the ages of 18-65 years, with a history of having been a psychiatric inpatient on an adult acute mental health ward during the study time period (6th March 2019 to 31st December 2020). The inclusion and exclusion criteria are summarized in Table 3. Out of these 200 patients undergoing Phase 1 testing, 40 will transition to Phase 2 of the study. The Phase 2 patient group will consist of 25±5 non-ID patients, and 15±5 patients with ID.

Study participants will be recruited from two inpatient units based in Leicestershire, UK. These units serve a population of approximately 1 million people living in both urban and rural areas within Leicester, Leicestershire and Rutland.[43] Applications to extend the study to further sites are currently in progress.

Patient and Public Involvement

The study design was refined following consultation group sessions with both patients with ID and their carers, as well as patients without ID and their carers. Significant changes made to the study design were made as a result of these sessions, including reduction of the questionnaire and interview burden, as well as incorporation of a stigma measure for patients with ID, as this was considered a research priority by patients with ID and their carers.

Patients and carers will all receive a report summarising the findings of the study, unless they request not to, and an easy-read version of the report will be developed for patients with ID.

Measures

The measures to be used are summarized in Table 4. The Phase 1 measures, which will be used to test non-ID participants only, include the AQ, [41] which is recommended by the National Institute for Health and Care Excellence as referral tools for adults with possible ASD who do not have ID.[44] Also included in the test battery are ASD tools which are less comprehensively validated in adult populations, but appear to show promise, including the Informant version of the Social Responsiveness Scale, 2nd edition[45] and the Self-report and Informant versions of the Adult Social Behaviour Questionnaire, ASBQ.[46] Phase 1 also includes a basic information form for participants, including demographic details, encompassing basic demographic information, and information pertaining to mental and physical health, including the 2014 APMS mental illness and physical health conditions checklists, thus enabling measurement harmonisation and wider comparison.[13] Additionally, both participants and informants will complete the EuroQol-5D-5L,[47] a self-report instrument that measures general health.

The Phase 2 measures include the DISCO[48] (though the interview will be limited to the diagnostic algorithm, psychiatric conditions and forensic problems items) and ADOS-2,[49]

For peer review only - http://bmjopen.bmj.com/site/about/guidelines.xhtml

both of which are tools with established validity for the diagnosis of ASDs. Also, psychiatric and physical comorbidities will be assessed using the relevant sections of the 2014 APMS interview.[13] Other tests include the field testing of the ASD interview of version 3 of the Schedules for Clinical Assessment in Neuropsychiatry, as well as the Stigma Questionnaire for people with Intellectual Disability.[50]

Outcome Parameters

The primary outcome parameter for this pilot study is the presence or absence of meeting ICD-10-DCR and DSM-5 diagnostic criteria for ASDs respectively. Secondary outcome parameters, including the study phase in which the corresponding parameters are collected, are summarised in Table 5.

Data Analysis Plan

The primary outcome parameter will be based on patients who transition to Phase 2 of the study. The ASD prevalence estimate will be calculated based on the proportion of Phase 2 participants who meet the ICD-10-DCR and DSM-5 diagnostic criteria for ASD on clinical assessment, with adjustment for selection and non-response, as well as the different study designs for the ID and non-ID patient subgroups.

For the feasibility study, the analyses will be descriptive. For continuous variables, values and standard deviations will be reported; for categorical variables, percentages and standard deviations will be reported. We will use multiple imputation to account for any missing data. For the full-powered study, the generalised linear model will be used to estimate the difference between groups, as well as corresponding OR, 95% CI and p-values.

The analyses will be primarily conducted in SPSS, but for more advanced statistical analyses (such as use of the generalised linear model), and graphical representation, we will use R.

Sample Size Justification

The sample size was calculated with direct statistician support, as well as the use of a statistical textbook and related software.[51] For a significance level of 0.05 and power of 85%, assuming estimates of 1% community ASD prevalence and 5% inpatient ASD prevalence,[15] a sample size of 374 patients is required. Ideally, we would be subjecting a minimum of 374 patients to Phase 2 testing. This is because only participants completing Phase 2 interview assessments will yield data pertaining to the primary outcome variable (i.e. the presence of absence of satisfying diagnostic criteria for ASD).

However, in the first instance, around 40 patients (from the minimum of 200 subjected to Phase 1 testing) will be selected for Phase 2 testing (via stratified random sampling for non-ID patients, whereas ID patients will progress automatically to Phase 2). The Phase 1 sample size of 200 patients takes into account the probability sampling method for Phase 2 selection, in addition to allowing for losses to follow-up for Phase 1 participants invited for Phase 2 testing. Our results will assume that the data for these 40 patients is representative of the larger group subjected to Phase 1 testing. Owing to the sample size, the SPRINT study should be considered a pilot/feasibility study, the intention of which being to establish and further refine the technical, administrative and logistical aspects of the study, with a view towards sample size expansion upon its completion. The sample size represents an appropriate sample size for a feasibility study of this type.[52,53]

Ethics and Dissemination

The written approval of the National Health Service Health Research Authority has been obtained, as well as the local ethics committee of the study centre. Written informed consent is and will be obtained from every participant. Significant modifications to the study protocol will be communicated to relevant members of the research team. Any results from this study will be published in peer-reviewed journals and conference proceedings. Additionally, the study's findings will be disseminated to all participants, including easy-read versions of the findings for those with intellectual disabilities.

Acknowledgements

The authors would like to acknowledge the following individuals for their contributions to the study. Nicola Spiers, Maria Viskaduraki and Zoe Morgan for statistical support. Elizabeta Mukaetova-Ladinska and Verity Chester for assistance with the systematic reviews underpinning the study. Dave Clarke and Henry Simkins for assistance with study design. Natalie Marking, Hannah Harrison, Tom Pringle, Deborah Glancy, Joanna McGarr, Rebekah Pole, Kris Roberts and Sam Pollen for support with study recruitment. Debra Bugler, Janice Holmes, Joy Fellows and Robin Oxley-Boyle for administrative support. Joanne Prosser and Naishali Chandarana for advice and support in developing easy-read materials. Freya Tyrer and Lesley Thoms for assistant with Patient and Public Involvement Sessions. The members of the Peoples Forum (Leicester, United Kingdom) and patients and carers at Leicestershire Partnership NHS Trust for providing valued input in the Patient and Public Involvement Sessions.

Contributor Statement

ST, TB, GY, RK, RA and MA-U were involved in drafting the study protocol. TB and ST conceived of the idea for this study. ST is the principal investigator for the study, under the

supervision of TB. ST led in writing the manuscript, TB and GY contributed revisions, and all authors approved the final manuscript.

Competing Interests

TB is directly involved with the development of the ASD interview questions for the Schedules for Assessment in Neuropsychiatry version 3, which have not yet been formally published; this study is involved in testing the clinical utility of these questions. Additionally, TB and RK were authors of the 2014 APMS, from which two questionnaires are being used. Neither TB nor RK will be receiving any monetary payment as a result of the inclusion of the ASD interview questions or the 2014 APMS questionnaires within this study.

Funding

This study was supported by funding awarded by an NIHR Senior Investigator, as well as receiving funding and resource support from the NIHR, as a result of the study being adopted onto the NIHR Clinical Research Network Portfolio of studies.

Data Sharing Statement

Deidentified participant data will be made available upon reasonable request from Sam Tromans, contactable on sjt56@leicester.ac.uk. The time period for availability would be within 6 months of journal publication of the study findings. Protocols and statistical analysis plans would be similarly available.

REFERENCES

1. World Health Organization. *The ICD-10 classification of mental and behavioural disorders: Clinical descriptions and diagnostic guidelines.* World Health Organization: Geneva, 1992.

2. Howlin P. Outcome in adult life for more able individuals with autism or asperger syndrome. *Autism*. 2000;4:63-83.

3. Newschaffer CJ, Croen LA, Daniels J, et al. The epidemiology of autism spectrum disorders. *Annu Rev Public Health*. 2007;28:235-258.

4. World Health Organization. *The ICD-10 classification of mental and behavioural disorders: Diagnostic criteria for research.* World Health Organization: Geneva, 1993.

5. American Psychiatric Association. *Diagnostic and statistical manual of mental disorders: DSM-5.* American Psychiatric Publishing: Arlington, 2013.

6. Smith IC, Reichow B, Volkmar FR. The effects of DSM-5 criteria on number of individuals diagnosed with autism spectrum disorder: A systematic review. *J Autism Dev Disord*. 2015;45:2541-2552.

7. MacLeod A, Guldberg K, Kossyvaki L, *et al*. Mind the gap: What is missing in the autism research agenda? https://www.birmingham.ac.uk/research/perspective/autism-research-experts.aspx. (Accessed 12 July 2019).

8. Lever AG, Geurts HM. Psychiatric co-occurring symptoms and disorders in young, middle-aged, and older adults with autism spectrum disorder. *J Autism Dev Disord*. 2016;46:1916-1930.

For peer review only

9. Kanner L. Early infantile autism. *J Pediatr*. 1944; 25: 211-217.

10. Wing L, Potter D. The epidemiology of autistic spectrum disorders: Is the prevalence rising? *Dev Disabil Res Rev*. 2002;8:151-161.

11. Baxter A, Brugha T, Erskine H, *et al*. The epidemiology and global burden of autism spectrum disorders. *Psychol Med*. 2015;45:601-613.

12. Brugha TS, Spiers N, Bankart J, *et al*. Epidemiology of autism in adults across age groups and ability levels. *Br J Psychiatry*. 2016;209:498-503.

13. McManus S, Bebbington P, Jenkins R, *et al*. *Mental health and wellbeing in England: Adult psychiatric morbidity survey 2014*. NHS Digital: Leeds, 2016.

14. Baird G, Simonoff E, Pickles A, *et al*. Prevalence of disorders of the autism spectrum in a population cohort of children in south thames: The special needs and autism project (SNAP). *Lancet*. 2006;368:210-215.

15. Tromans S, Chester V, Kiani R, *et al*. The prevalence of autism spectrum disorders in adult psychiatric inpatients: A systematic review . *Clin Pract Epidemiol Ment Health*. 2018; 14: 177-187.

16. Mandell DS, Lawer LJ, Branch K, *et al*. Prevalence and correlates of autism in a state psychiatric hospital. *Autism*. 2012;16:557-567.

17. Shah A, Holmes N, Wing L. Prevalence of autism and related conditions in adults in a mental handicap hospital. *Appl Res Ment Retard*. 1982;3:303-317.

18. Scragg P, Shah A. Prevalence of asperger's syndrome in a secure hospital. *Br J Psychiatry*. 1994;165:679-682.

For peer review only

19. Hare DJ, Gould J, Mills R, *et al*. *A preliminary study of individuals with autistic spectrum disorders in three special hospitals in England.* National Autistic Society: London, 1999.

20. Ghaziuddin M, Zafar S. Psychiatric comorbidity of adults with autism spectrum disorders. *Clin Neuropsychiatry*. 2008;5:9-12.

21. Simonoff E, Pickles A, Charman T, *et al*. Psychiatric disorders in children with autism spectrum disorders: Prevalence, comorbidity, and associated factors in a population-derived sample. *J Am Acad Child Adolesc Psychiatry*. 2008;47:921-929.

22. de Bruin EI, Ferdinand RF, Meester S, *et al.* High rates of psychiatric co-morbidity in PDD-NOS. *J Autism Dev Disord*. 2007;37:877-886.

23. Mattila M, Hurtig T, Haapsamo H, *et al.* Comorbid psychiatric disorders associated with asperger syndrome/high-functioning autism: A community-and clinic-based study. *J Autism Dev Disord*. 2010;40:1080-1093.

24. Leyfer OT, Folstein SE, Bacalman S, *et al.* Comorbid psychiatric disorders in children with autism: Interview development and rates of disorders. *J Autism Dev Disord*. 2006;36:849-861.

25. Brereton AV, Tonge BJ, Einfeld SL. Psychopathology in children and adolescents with autism compared to young people with intellectual disability. *J Autism Dev Disord*. 2006;36:863-870.

26. Cooper SA, Smiley E, Morrison J, *et al*. Mental ill-health in adults with intellectual disabilities: Prevalence and associated factors. *Br J Psychiatry*. 2007;190:27-35.

For peer review only

27. Joshi G, Wozniak J, Petty C, *et al.* Psychiatric comorbidity and functioning in a clinically referred population of adults with autism spectrum disorders: A comparative study. *J Autism Dev Disord*. 2013;43:1314-1325.

28. Howlin P, Goode S, Hutton J *et al*. Adult outcome for children with autism. *J Child Psychol Psychiatry*. 2004;45:212-229.

29. Cascio MT, Cella M, Preti A, *et al*. Gender and duration of untreated psychosis: A systematic review and meta-analysis. *Early Interv Psychiatry*. 2012;6:115-127.

30. Cashin A, Buckley T, Trollor JN, *et al*. A scoping review of what is known of the physical health of adults with autism spectrum disorder. *J Intellect Disabil*. 2016;22:96-108.

31. Eaves LC, Ho HH. Young adult outcome of autism spectrum disorders. *J Autism Dev Disord*. 2008;38:739-747.

32. Ho HH, Eaves LC, Peabody D. Nutrient intake and obesity in children with autism. *Focus Autism Other Dev Disabl*. 1997;12:187-192.

33. Granich J, Lin A, Hunt A, *et al*. Obesity and associated factors in youth with an autism spectrum disorder. *Autism*. 2016;20:916-926.

34. Levy SE, Giarelli E, Lee LC, *et al.* Autism spectrum disorder and co-occurring developmental, psychiatric, and medical conditions among children in multiple populations of the united states. *J Dev Behav Pediatr*. 2010;31:267-275.

35. Schieve LA, Gonzalez V, Boulet SL, *et al.* Concurrent medical conditions and health care use and needs among children with learning and behavioral developmental disabilities, national health interview survey, 2006–2010. *Res Dev Disabil*. 2012;33:467-476.

For peer review only

36. Bauman ML. Medical comorbidities in autism: Challenges to diagnosis and treatment. *Neurotherapeutics*. 2010;7:320-327.

37. Muskens JB, Velders FP, Staal WG. Medical comorbidities in children and adolescents with autism spectrum disorders and attention deficit hyperactivity disorders: A systematic review. *Eur Child Adolesc Psychiatry*. 2017;26:1093-1103.

38. Croen LA, Zerbo O, Qian Y, *et al.* The health status of adults on the autism spectrum. *Autism*. 2015;19:814-823.

39. Brugha T, Tyrer F, Leaver A, *et al.* Testing adults by questionnaire for social and communication disorders, including autism spectrum disorders, in an adult mental health service population. *Int J Methods Psychiatr Res*. In press.

40. Ferriter M, Hare D, Bendall P, *et al.* Brief report: Assessment of a screening tool for autistic spectrum disorders in adult population. *J Autism Dev Disord*. 2001;31:351-353.

41. Baron-Cohen S, Wheelwright S, Skinner R, *et al*. The autism-spectrum quotient (AQ): Evidence from asperger syndrome/high-functioning autism, males and females, scientists and mathematicians. *J Autism Dev Disord*. 2001;31:5-17.

42. National Institute for Health and Care Excellence. Autism spectrum disorder in adults: Diagnosis and management (CG142). https://www.nice.org.uk/guidance/cg142/resources/autism-spectrum-disorder-in-adults-diagnosis-and-management-pdf-35109567475909 (Accessed 12 July 2019).

43. Leicestershire Partnership NHS Trust. Basic facts and figures. . http://www.leicspart.nhs.uk/_Aboutus-WhoWeAreandWhatWeDo-FactsandFigures.aspx (Accessed 12 July 2019).

For peer review only

44. National Institute for Health and Care Excellence. Autism spectrum disorder in adults: Diagnosis and management. NICE guideline [CG142]. 2012.

45. Constantino JN, Gruber CP. *Social responsiveness scale (SRS).* Western Psychological Services: California, 2012.

46. Horwitz E, Schoevers R, Ketelaars C, *et al.* Clinical assessment of ASD in adults using self-and other-report: Psychometric properties and validity of the adult social behavior questionnaire (ASBQ). *Res Autism Spectr Disord*. 2016;24:17-28.

47. Herdman M, Gudex C, Lloyd A, *et al.* Development and preliminary testing of the new five-level version of EQ-5D (EQ-5D-5L). *Qual Life Res*. 2011;20:1727-1736.

48. Wing L, Leekam SR, Libby SJ, *et al*. The diagnostic interview for social and communication disorders: Background, inter-rater reliability and clinical use. *J Child Psychol Psychiatry*. 2002;43:307-325.

49. Lord C, Rutter M, DiLavore P *et al*. *Autism diagnostic observation schedule–2nd edition (ADOS-2).* Western Psychological Corporation: California, 2012.

50. Ali A, Strydom A, Hassiotis A, *et al*. A measure of perceived stigma in people with intellectual disability. *Br J Psychiatry*. 2008;193:410-415.

51. Machin D, Campbell MJ, Tan S, *et al*. *Sample size tables for clinical studies.* Wiley-Blackwell: New Jersey, 2011.

52. Johanson GA, Brookes, GP. Initial scale development: sample size for pilot studies. *Educ Psychol Meas*. 2010;70:394-400.

For peer review only

53. Hertzog MA. Considerations in determining sample size for pilot studies. *Res Nurs Health.* 2008;31:180-191.

For peer review only

Figure 1: Flow chart summary of study design.

For peer review only

Table 1: Summaries of the ICD-10-DCR[4] and DSM-5[5] diagnostic criteria for ASD. For the diagnostic criteria in full, please refer to the source texts.

**ICD-10-DCR Criteria for Childhood Autism[3]:**<br>A. Presence of abnormal or impaired development before the age of three years<br>B. Qualitative abnormalities in reciprocal social interaction<br>C. Qualitative abnormalities in communication<br>D. Restricted, repetitive, and stereotyped patterns of behaviour, interests and activities<br>E. The clinical picture is not attributable to the other varieties of pervasive developmental disorder; specific developmental disorder of receptive language with secondary socio-emotional problems; reactive attachment disorder or disinhibited attachment disorder; mental retardation with some associated emotional or behavioural disorder; schizophrenia of unusually early onset; and Rett's syndrome.
**DSM-5 Criteria for Autism Spectrum Disorder:**<br>A. Persistent deficits in social communication and social interaction across multiple contexts<br>B. Restricted, repetitive patterns of behaviour, interests, or activities<br>C. Symptoms must be present in the early developmental period<br>D. Symptoms cause clinically significant impairment in social, occupational or other important areas of current functioning<br>E. These disturbances are not better explained by intellectual disability (ID)[4]

[3] ICD-10-DCR also recognises the diagnosis of 'atypical autism', whereby the features of abnormal development only manifest for the first time after 3 years of age (A) and/or the affected individual does not satisfy all three of the clinical diagnostic criteria (B-D).
[4] DSM-5 recognises that ASD and ID frequently co-exist, but that "to make comorbid diagnoses of autism spectrum disorder and intellectual disability, social communication should be below that expected for general developmental level."

For peer review only

Table 2: The probability of selection for Phase 2 according to total scores in the AQ questionnaire. This probability framework has been validated in a previous study conducted on adult participants from a mixture of inpatient and community mental health settings.[39] Participants scoring higher on these questionnaires will have a greater probability of selection.

AQ score	Probability of selection
≤19	0.1
20-24	0.2
25-29	0.3
30-39	0.6
≥40	1.0

For peer review only

Table 3: Inclusion and exclusion criteria. Additionally, participants lacking capacity will be withdrawn from the study if they become distressed by the assessment process and/or their guardians are not in agreement with them remaining in the study.

Inclusion Criteria	Exclusion Criteria
Aged between 18-65 years.	<18 or >65 years of age
Being or having been a psychiatric inpatient on an adult acute mental health ward during the study time period.	Having no history of being a psychiatric inpatient on an adult acute mental health ward during the study time period.
	Clinical diagnosis of dementia.
	Not understanding written and/or verbal English

For peer review only

Table 4: Summary of study measures

Measure	Purpose
**Phase 1** (Non-ID participants only)	
Autism Quotient, AQ[41]	Measuring likelihood of ASD.
Informant version of the Social Responsiveness Scale,2nd edition, SRS-A[45]	
Self-report and Informant versions of the Adult Social Behaviour Questionnaire, ASBQ[46]	
Self-report and Informant versions of the EuroQol-5D-5L, EQ-5D-5L[47]	Measuring quality of life.
Basic information form	Collecting information pertaining to patient demographics, as well as the 2014 Adult Psychiatric Morbidity Survey (APMS) mental illness and physical health conditions checklists[13]
**Phase 2**	
Diagnostic Interview for Social and Communication Disorders, DISCO[48]	Establishing whether participant meets diagnostic criteria for ASDs.
Autism Diagnostic Observation Schedule version 2, ADOS-2[49]	
The Mental Illness Diagnoses and Treatment section of the 2014 APMS[13]	Establishing participant's psychiatric and physical health comorbidities.
The Physical Health Conditions section of the 2014 APMS[13]	
ASD interview subsection of version 3 of the Schedules for Clinical Assessment in Neuropsychiatry, ASD-SCAN-3	Field-testing of interview schedule (for non-ID participants only)
Stigma Questionnaire for people with Intellectual Disability, SQID[50]	Participants experience of stigma (for ID participants only)
Basic information form	Collecting information pertaining to patient demographics, as well as the 2014 Adult Psychiatric Morbidity Survey (APMS) mental illness and physical health conditions checklists[13] (for ID participants only, as this form is completed in Phase 1 for non-ID participants).

Table 5: Summary of secondary outcome parameters

Outcome Category	Corresponding Parameters	Study Phase in which Data is Collected
**Autism questionnaire data**	• Autism Quotient, AQ[41]<br>• Informant version of the Social Responsiveness Scale, 2nd edition, SRS-A[45]<br>• Self-report and Informant versions of the Adult Social Behaviour Questionnaire, ASBQ[46]	• Phase 1
**Basic demographic information**	• Date of birth<br>• Sex<br>• Postcode<br>• Ethnic group<br>• Employment status<br>• Relationship status	• Phase 1 (non-ID participants)<br>• Phase 2 (ID participants)
**Mental health history**	• Date of most recent psychiatric hospital admission<br>• Discharge date of most recent psychiatric hospital admission (where applicable)<br>• Total number of inpatient psychiatric admissions<br>• 2014 Adult Psychiatric Morbidity Survey (APMS) mental illness checklist[13]	• Phase 1 (non-ID participants)<br>• Phase 2 (ID participants)
	• The Mental Illness Diagnoses and Treatment section of the 2014 APMS[13]	• Phase 2
**Physical health history**	• 2014 APMS physical health conditions checklist[13]	• Phase 1 (non-ID participants)<br>• Phase 2 (ID participants)
	• The Physical Health Conditions section of the 2014 APMS[13]	• Phase 2
**General health**	• EuroQol-5D-5L[47] data for participants and informants	• Phase 1
**Stigma experience (ID participants only)**	• Stigma Questionnaire for people with Intellectual Disability, SQID[50]	• Phase 2 (ID participants)

For peer review only

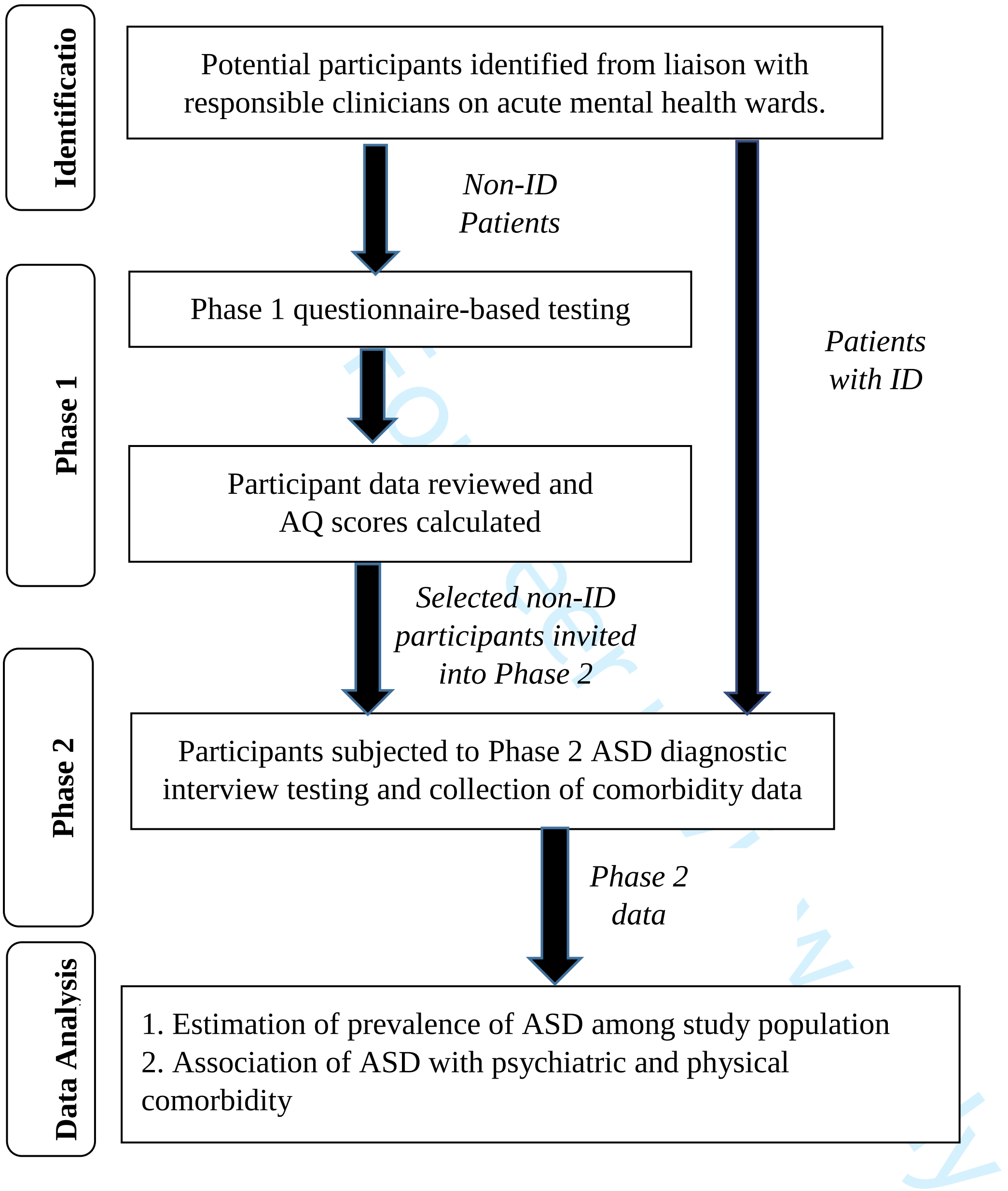
Identificatio
Potential participants identified from liaison with responsible clinicians on acute mental health wards.
Non-ID Patients
Patients with ID
Phase 1
Phase 1 questionnaire-based testing
Participant data reviewed and AQ scores calculated
Selected non-ID participants invited into Phase 2
Phase 2
Participants subjected to Phase 2 ASD diagnostic interview testing and collection of comorbidity data
Phase 2 data
Data Analysis
1. Estimation of prevalence of ASD among study population
2. Association of ASD with psychiatric and physical comorbidity

STROBE Statement—checklist of items that should be included in reports of observational studies

	Item No	Recommendation
**Title and abstract**	1	(*a*) Indicate the study's design with a commonly used term in the title or the abstract
		(*b*) Provide in the abstract an informative and balanced summary of what was done and what was found — X n/a (study protocol)
**Introduction**		
Background/rationale	2	Explain the scientific background and rationale for the investigation being reported
Objectives	3	State specific objectives, including any prespecified hypotheses
**Methods**		
Study design	4	Present key elements of study design early in the paper
Setting	5	Describe the setting, locations, and relevant dates, including periods of recruitment, exposure, follow-up, and data collection
Participants	6	(*a*) *Cohort study*—Give the eligibility criteria, and the sources and methods of selection of participants. Describe methods of follow-up<br>*Case-control study*—Give the eligibility criteria, and the sources and methods of case ascertainment and control selection. Give the rationale for the choice of cases and controls<br>*Cross-sectional study*—Give the eligibility criteria, and the sources and methods of selection of participants
		(*b*) *Cohort study*—For matched studies, give matching criteria and number of exposed and unexposed<br>*Case-control study*—For matched studies, give matching criteria and the number of controls per case
Variables	7	Clearly define all outcomes, exposures, predictors, potential confounders, and effect modifiers. Give diagnostic criteria, if applicable
Data sources/ measurement	8*	For each variable of interest, give sources of data and details of methods of assessment (measurement). Describe comparability of assessment methods if there is more than one group
Bias	9	Describe any efforts to address potential sources of bias
Study size	10	Explain how the study size was arrived at
Quantitative variables	11	Explain how quantitative variables were handled in the analyses. If applicable, describe which groupings were chosen and why
Statistical methods	12	(*a*) Describe all statistical methods, including those used to control for confounding
		(*b*) Describe any methods used to examine subgroups and interactions
		(*c*) Explain how missing data were addressed
		(*d*) *Cohort study*—If applicable, explain how loss to follow-up was addressed<br>*Case-control study*—If applicable, explain how matching of cases and controls was addressed<br>*Cross-sectional study*—If applicable, describe analytical methods taking account of sampling strategy
		(*e*) Describe any sensitivity analyses

Continued on next page

**Results**		
Participants	13*	(a) Report numbers of individuals at each stage of study—eg numbers potentially eligible, examined for eligibility, confirmed eligible, included in the study, completing follow-up, and analysed
		(b) Give reasons for non-participation at each stage
		(c) Consider use of a flow diagram
Descriptive data	14*	(a) Give characteristics of study participants (eg demographic, clinical, social) and information on exposures and potential confounders
		(b) Indicate number of participants with missing data for each variable of interest
		(c) *Cohort study*—Summarise follow-up time (eg, average and total amount)
Outcome data	15*	*Cohort study*—Report numbers of outcome events or summary measures over time
		Case-control study—Report numbers in each exposure category, or summary measures of exposure
		Cross-sectional study—Report numbers of outcome events or summary measures
Main results	16	(*a*) Give unadjusted estimates and, if applicable, confounder-adjusted estimates and their precision (eg, 95% confidence interval). Make clear which confounders were adjusted for and why they were included
		(*b*) Report category boundaries when continuous variables were categorized
		(*c*) If relevant, consider translating estimates of relative risk into absolute risk for a meaningful time period
Other analyses	17	Report other analyses done—eg analyses of subgroups and interactions, and sensitivity analyses
**Discussion**		
Key results	18	Summarise key results with reference to study objectives
Limitations	19	Discuss limitations of the study, taking into account sources of potential bias or imprecision. Discuss both direction and magnitude of any potential bias
Interpretation	20	Give a cautious overall interpretation of results considering objectives, limitations, multiplicity of analyses, results from similar studies, and other relevant evidence
Generalisability	21	Discuss the generalisability (external validity) of the study results
**Other information**		
Funding	22	Give the source of funding and the role of the funders for the present study and, if applicable, for the original study on which the present article is based

*Give information separately for cases and controls in case-control studies and, if applicable, for exposed and unexposed groups in cohort and cross-sectional studies.

Note: An Explanation and Elaboration article discusses each checklist item and gives methodological background and published examples of transparent reporting. The STROBE checklist is best used in conjunction with this article (freely available on the Web sites of PLoS Medicine at http://www.plosmedicine.org/, Annals of Internal Medicine at http://www.annals.org/, and Epidemiology at http://www.epidem.com/). Information on the STROBE Initiative is available at www.strobe-statement.org.

For peer review only - http://bmjopen.bmj.com/site/about/guidelines.xhtml
